# Garvicin Q: characterization of biosynthesis and mode of action

**DOI:** 10.1186/s12934-022-01952-9

**Published:** 2022-11-11

**Authors:** Christian K. Desiderato, Katharina M. Hasenauer, Sebastian J. Reich, Oliver Goldbeck, Lalaina Holivololona, Kirill V. Ovchinnikov, Alexander Reiter, Marco Oldiges, Dzung B. Diep, Bernhard J. Eikmanns, Christian U. Riedel

**Affiliations:** 1 Institute of Microbiology and Biotechnology, University of Ulm, Albert-Einstein-Allee 11, 89081 Ulm, Germany; 2 Faculty of Chemistry, Biotechnology and Food Science, Norwegian University of Life Sciences, Ås, Norway; 3 Institute of Bio- and Geosciences, Forschungszentrum Jülich GmbH, IBG-1: Biotechnology, 52425 Jülich, Germany; 4 Institute of Biotechnology, RWTH Aachen University, 52062 Aachen, Germany

## Abstract

Bacteriocins are ribosomally synthesized antimicrobial peptides, that either kill target bacteria or inhibit their growth. Bacteriocins are used in food preservation and are of increasing interest as potential alternatives to conventional antibiotics. In the present study, we show that *Lactococcus petauri* B1726, a strain isolated from fermented balsam pear, produces a heat-stable and protease-sensitive compound. Following genome sequencing, a gene cluster for production of a class IId bacteriocin was identified consisting of *garQ* (encoding for the bacteriocin garvicin Q), *garI* (for a putative immunity protein), *garC*, and *garD* (putative transporter proteins). Growth conditions were optimized for increased bacteriocin activity in supernatants of *L. petauri* B1726 and purification and mass spectrometry identified the compound as garvicin Q. Further experiments suggest that garvicin Q adsorbs to biomass of various susceptible and insusceptible bacteria and support the hypothesis that garvicin Q requires a mannose-family phosphotransferase system (PTS^Man^) as receptor to kill target bacteria by disruption of membrane integrity. Heterologous expression of a synthetic *garQICD* operon was established in *Corynebacterium glutamicum* demonstrating that genes *garQICD* are responsible for biosynthesis and secretion of garvicin Q. Moreover, production of garvicin Q by the recombinant *C. glutamicum* strain was improved by using a defined medium yet product levels were still considerably lower than with the natural *L. petauri* B1726 producer strain.

Collectively, our data identifies the genetic basis for production of the bacteriocin garvicin Q by *L. petauri* B1726 and provides insights into the receptor and mode of action of garvicin Q. Moreover, we successfully performed first attempts towards biotechnological production of this interesting bacteriocin using natural and heterologous hosts.

**Supplementary Information:**

The online version contains supplementary material available at 10.1186/s12934-022-01952-9.

## Introduction

Hyperacute haemorrhagic septicaemia, also termed lactococcosis, is a disease of fish species that is characterized by high mortality rates, and thus leads to economic losses in aquaculture [1, 2]. The closely related species *Lactococcus garvieae* and *Lactococcus petauri* were identified as the etiological agents of this disease in rainbow trout (*Oncorhynchus mykiss*) and grey mullet (*Mugil cephalus*) [3–6]. *L. garvieae* infections have also been reported for the crustacean giant freshwater prawn *Macrobrachium rosenbergii* [7]. Moreover, there is an increasing number of reports on *L. garvieae* as an emerging zoonotic pathogen infecting ruminants and humans [1, 8]. Infections of humans by *L. garvieae* are often associated with contact to contaminated raw fish [9, 10]. Interestingly, *L. garvieae* and *L. petauri* strains are widely distributed in the environment and were isolated not only from fish but also from various food products including raw milk, artisan cheese and vegetables [8, 11–14].

Bacterial infections of fish are traditionally treated or prevented by antibiotics and thus it is not surprising that antibiotic resistances are widespread amongst pathogens associated with seafood [15, 16]. There are numerous reports on *L. garvieae* isolates with (multiple) resistances towards clinically relevant antibiotics like erythromycin and tetracycline [1]. As alternatives to conventional antibiotics, vaccination of fish, bacteriophages or probiotic bacteria have been investigated for their potential to combat *L. garvieae* infection [17–19]. Another interesting approach is the application of antimicrobial peptides produced by bacteria, so called bacteriocins, in aquaculture systems [20].

Bacteriocins are ribosomally synthesized antimicrobial peptides that directly kill target bacteria or inhibit their growth [21] and provide the producing organisms with competitive advantages over other bacteria in their ecological niche [22]. *L. garvieae* strains have also been described to produce various bacteriocins including garvicin Q [23], garvicin KS [24], garvicin A, B and C [25, 26], garvicin ML [28], garviecin LG34 [29] and garviecin L1-5 [30]. Garvicins KS, Q, and ML and garviecin L1-5 have a broad antimicrobial spectrum, whereas garvicin A, B and C mainly kill *L. garvieae* strains. Hence, these bacteriocins are interesting candidates to treat lactococcosis. Recently, it has been demonstrated that garvicin KS is a promising candidate for protection of fish against *L. garvieae* infection [31]. Low concentrations of garvicin KS exhibited no cytotoxicity to fish cell lines and significantly increased survival of zebrafish larvae upon *L. garvieae* challenge, highlighting the potential of bacteriocins for infection control in aquacultures [31]. However, application of bacteriocins is limited by a lack of knowledge about the characteristics of the peptides and by expensive production of pure peptide [21].

In the present study, we identified *L. petauri* B1726 as garvicin Q producer, analysed the genetic locus for garvicin Q, characterised its mode of action, and investigated conditions for its production by *L.* *petauri* B1726 and *C. glutamicum*.

## Material and methods

### Strains and growth conditions

*L. petauri* B1726 was routinely cultivated in MRS medium unless stated otherwise. For optimization of garvicin Q production*, L. petauri* B1726 was cultivated statically in M17, 2xTY or BHI medium with 2% (w/v) glucose or lactose at 30 or 37 °C in glass tubes or Schott glass bottles as indicated in figure legends. Anaerobic cultivations were performed in a Millipore anaerobic jar with Oxoid AnaeroGen bags. *Listeria* spp*.* were cultivated in BHI medium at 37 °C under shaking conditions (130 rpm). *Lactococcus* *lactis* IL1403 was grown in M17 medium with 0.5% (w/v) glucose (GM17) overnight at 30 °C under shaking conditions (130 rpm). *C. glutamicum* strains were cultivated overnight in 2xTY at 30 °C under shaking conditions (130 rpm). For recombinant garvicin Q synthesis, *C. glutamicum* CR099/pPBEx2-*garQICD*^*Cgl*^ was cultivated in 2xTY with 2% (w/v) glucose or CGXII minimal medium [32] with 2% (w/v) glucose. Media were supplemented with 25 µg kanamycin ml^−1^ (*C. glutamicum*), 10 µg chloramphenicol ml^−1^ (*Listeria* spp. and *L. lactis*) and/or 0.2 mM IPTG (*C. glutamicum*) as appropriate. All strains are listed in Table 1.

**Table 1:** Bacterial strains used in the present study

Strain	Relevant characteristics	Source
*L. petauri B1726*	Isolated garvicin Q producer	This study
*L. innocua*		
LMG2785	Garvicin Q sensor strain	[33]
LMG2785/pNZ-pHin2^*Lm*^	LMG2785 derivative harbouring the pNZ-pHin2^*Lm*^ plasmid for constitutive expression of pHluorin2	[34]
*L. monocytogenes*		
EGDe	Type strain, serotype 1/2a	[35]
EGY2	EGDe derivative containing a 84 bp deletion in the *mptD* gene	[36]
*L. lactis*		
IL1403	Lab strain, plasmid-free derivative of strain IL594 isolated from a cheese starter culture	[37]
IL1403/pNZ-pHin2^*Lm*^	IL1403 derivative harbouring the pNZ-pHin2^*Lm*^ plasmid for constitutive expression of pHluorin2	This study
* E. coli DH5α*	Cloning host	[38]
*C. glutamicum*		
ATCC13032	Wild type strain	ATCC^a^
CR099	ATCC13032 derivative; cured of prophages CGP1, CGP2 and CGP3 and of insertion elements ISCg1 and ISCg2	[39]
CR099/pPBEx2	CR099 harbouring the expression vector pPBEx2	This study
CR099/pPBEx2-*garQICD*^***Cgl***^	CR099 harbouring the plasmid pPBEx2-*garQICD*^*Cgl*^ for IPTG-inducible expression of codon optimized garvicin Q biosynthesis genes	This study

^a^*ATCC* american type culture collection

### Genome sequencing and annotation

Whole genome sequencing of *L. petauri* B1726 was performed using Illumina and Oxford Nanopore Technology (ONT) sequencing as follows. High molecular weight gDNA was prepared from a MRS overnight culture of *L. petauri* B1726 using the MagAttract HMW DNA kit (Qiagen) according to the manufacturer’s instructions for Gram-positive bacteria. Library preparation was done using the rapid barcoding kit SQK-RBK004 (ONT). ONT sequencing was carried out for 24 h using a FLO-FLG001 flow cell on a MinION platform with Flongle adapter controlled by MinKNOW v. 21.10.4 (all ONT). Base calling of raw data was done using the super-accurate base calling model of the guppy algorithm (v. 6.0.1, ONT) on a CUDA-capable RTX 3070 GPU (Nvidia). *L. petauri* B1726 reads were then filtered using filtlong v. 0.2.1 with recommended settings [40]. The library for Illumina sequencing was prepared using the Nextera^™^ Flex Library Preparation kit (Illumina). Sequencing was performed on an Illumina MiSeq system. Illumina short reads were filtered before further processing using fastp (Chen et al. 2018). Coverages were 21 ×for ONT and 74 ×for Illumina data. Both, the ONT and the Illumina data sets were then passed to the hybrid assembly pipeline of Unicycler v. 0.5.0 [41], which resulted in a single circular contig of 2.106 Mega base pairs. Subsequent short read polishing was done using Polypolish v. 0.5.0 [42] and POLCA (part of MaSuRCA v. 4.0.8, [43]). Automated annotation and identification of rRNA and tRNA genes of the polished consensus sequence was done using prokka v. 1.14.6 [44]. The annotated version of the *L. petauri* B1726 genome has been submitted to GenBank (Accession Number: CP094882.1). Raw sequencing data is available on the NCBI Sequencing Read Archive with accession numbers SRR18554891 (ONT data) and SRR18554892 (Illumina data).

### Nucleotide and amino acid sequence analysis

The online tools BAGEL4 [45] and AntiSMASH version 6.0.1 [46] were used for prediction of bacteriocin gene clusters. Terminator and promoter prediction was done with ARNold [47], BPROM and FGENESB [48] online tools. JSpeciesWS version 3.9.0 [49] was used for whole genome sequence alignment. BLASTp [50] and Clustal Omega [51] was used for protein sequence alignment. Protter version 1.0 [52] and PPM 3.0 web server [53] were applied for prediction of membrane topology of selected proteins. To gain structural insights, deep learning modelling method RoseTTAFold of Robetta [54] was used for tertiary structure prediction. The complex of group I PTS^Man^ subunits IIC and IID and garvicin Q was predicted using AlphaFold [55]. Visualization of protein structures was done with ChimeraX [56].

### Molecular biology procedures

Cloning procedures were performed using standard reagents according to protocols of the manufacturers. Primers and codon-optimized genes were purchased from a commercial service provider (Eurofins Genomics) and sequences are listed in Additional file 1: Table S1. For construction of pPBEx2-*garQICD*^*Cgl*^, sequences of the *L.* *petauri* B1726 genes *garQ*, *garI*, *garC*, and *garD* were codon-optimized for *C. glutamicum*. The codon-optimized genes were amplified by PCR using Q5 high fidelity polymerase (New England Biolabs) and specific primers. The empty plasmid pPBEx2 [57] was linearized with *Pst*I and *Kpn*I and ligated to PCR products in an isothermal reaction [58]. *E. coli* DH5α was transformed with the assembled plasmid pPBEx2-*garQICD*^*Cgl*^ and the plasmid sequence was confirmed by Sanger sequencing by a commercial service provider (Microsynth Seqlab). Transformation of *C. glutamicum* CR099 with pPBEx2-*garQICD*^*Cgl*^ was performed according to a standard protocol [59]. Construction of pNZ-pHin2^*Lm*^ was described elsewhere [34] and transformation of *L. lactis* IL1403 was achieved using a standard protocol [60].

### Overlay agar plate assay

The overlay agar plates were prepared as described previously [61] with slight modifications. An appropriate dilution of a *L. petauri* B1726 overnight culture was spread on GM17 agar plates and immediately overlayed with fresh GM17 agar. After two days of incubation at 30 °C single colonies of *L. petauri* B1726 were observed. The sandwiched colonies were then overlayed with BHI agar containing *Listeria* *innocua* LMG2785 diluted to an optical density at 600 nm (OD_600_) of ~ 0.002. The agar plates were then incubated for one day at 30 °C and plates were imaged in an iBright^™^ FL1000 System.

### Growth inhibition assay

To determine the antimicrobial activity of garvicin Q-containing samples a standard microtiter plate assay [62] was performed with slight modifications. Two-fold serial dilutions of the sample were prepared with BHI or GM17. The indicator strains *L. innocua* LMG2785 or *L. lactis* IL1403 were cultivated overnight and diluted in fresh medium to an OD_600_ of 0.2 prior to the assay. Diluted samples (100 µl) were mixed with the indicator strain suspension (100 µl) in a sterile 96-well plate corresponding to a start OD_600_ of 0.1. The microtiter plate was incubated at 37 °C for 4–5 h with shaking and growth of the indicator strain was determined by measuring the OD_600_ in an Infinite M200 plate reader (Tecan). Bacteriocin activity was determined in a semi-quantitative manner as described previously [62]. Briefly, bacteriocin units per ml (BU ml^−1^) were calculated as the reciprocal of the highest dilution showing at least 50% inhibition of the indicator strain.

### pHluorin2 assay

The pHluorin2 assay was performed according to a previously described protocol using reporter strains *L. innocua* LMG2785/pNZ-pHin2^*Lm*^ [34] and *L. lactis* IL1403/pNZ-pHin2^*Lm*^ (this study). These reporter strains constitutively express the pH-sensitive fluorescent protein pHluorin2, which shows a bimodal excitation spectrum with maxima at 400 and 480 nm. If pH homoeostasis is disrupted by membrane-damaging compounds, intracellular pH drops to the level of the assay buffer (pH 6.2) and this leads to a ratiometric change in the fluorescence intensity of pHLuorin2 at the two excitation peaks. For assays, reporter strains were cultivated overnight, washed once with phosphate-buffered saline, and then resuspended in LMB buffer (pH 6.2) to an OD_600_ of 3. Two-fold serial dilutions of samples were prepared in a similar manner as described for the growth inhibition assay and 100 µl of sensor strain suspension was mixed with 100 µl of sample dilution. The mixture was incubated for 30 min in the dark at room temperature. Fluorescence intensities (emission at 520 nm) were determined with excitation at 400 and 480 nm. The ratios of emission intensities after excitation at 400 and 480 nm were calculated.

### Purification and identification of garvicin Q

First purifications of garvicin Q were performed following an established protocol [63]. The peptide was purified by ammonium sulphate (AS) precipitation, cation exchange chromatography (CIEX) and reversed-phase chromatography (RPC) as described previously [63]. Garvicin Q eluted at ~ 49% elution buffer during RPC.

For later purifications an optimized purification protocol including hydrophobic interaction chromatography (HIC) was applied. *L. petauri* B1726 was cultivated overnight at 30 °C in GM17 (2% glucose) and the culture supernatant (SN) was collected by centrifugation. Then, 0.64 M ammonium sulphate was slowly added to the SN and the pH was set to 6 to match conditions during HIC. The sample was then again centrifuged to remove insoluble components. The 1 ml HiTrap Octyl FF column (Cytiva) was equilibrated with 5 column volumes of 50 mM sodium phosphate buffer at pH 6 with 0.64 M ammonium sulphate. Then ~ 100 ml of the SN retained after AS precipitation was loaded onto the column and washed with 20 column volumes of equilibration buffer. Garvicin Q was eluted with 10 column volumes of HPLC water. Fractions containing bacteriocin activity (in total 4 ml) were subjected to RPC as described earlier [63] using acetonitrile as a mobile phase instead of 2-propanol. Garvicin Q eluted at ~ 30% elution buffer. The garvicin Q preparation was either stored at 4 °C or dried under vacuum for long-term storage.

Mass spectrometry analysis was performed based on the method described in [64]. Protein fractions were concentrated to 1 mg ml^−1^ for tryptic digestion with 1 μg trypsin in a total volume of 100 μl for 5 h at 42 °C as recommended by the supplier. Peptide solutions were diluted 1:2 with LC–MS-grade H_2_O prior LC–MS measurements.

Liquid chromatography mass spectrometry (LC–MS) was conducted with an Agilent 1260 Infinity system (Agilent Technologies, Waldbronn, Germany) coupled to a quadrupole time-of-flight mass spectrometer (TripleTOF6600, AB Sciex, Darmstadt, Germany). LC was performed with an Ascentis^®^ Express Peptide ES-C18, 2.7 μm HPLC column (53,307-U, Merck, Darmstadt, Germany) with a flow rate of 200 µL min^−1^ and the mobile phases (A) 0.1% formic acid in water and (B) acetonitrile. The elution gradient was as follows: 0 min, 3% B; 70 min, 40% B; 78 min 40% B, 79 min 60% B, 89 min 60% B, 90 min 3% B followed by a 12 min equilibration time between injections. Column temperature was set to 21 °C and injection volume to 10 µL. MS was conducted with a TurboV ion source operated in positive ionization mode. Ion spray voltage was set to 5.5 kV, source temperature to 450 °C, curtain gas to 35 psi, and the support gases GS1/GS2 to 50 psi/50psi. All gases were nitrogen. The quadrupole time-of-flight (QToF) mass spectrometer was operated in data-dependent acquisition (DDA) mode. Based on a ToF survey scan with a dwell time of 250 ms, product ion scans with a dwell-time of 100 ms were automatically performed for a maximum of 40 ions with a mass-to-charge ratio > 300 m/z, a charge of 2–4 passing an intensity threshold of 150 cps. After acquisition, mass-to-charge ratios were excluded from the potential candidate-ion list for 12 s. Declustering potential was set to 120 V, collision energy spread to 5 V and mass tolerance to 25 ppm. Acquired mass spectra were analyzed with PeakView 2.1 (AB Sciex, Darmstadt, Germany). Protein identification was performed with the ProteinPilot 5.1 software (AB Sciex, Darmstadt, Germany). Complete results of the MS analyses are provided in Additional files 2, 3.

### SDS-PAGE, silver staining and zymogram analysis

SDS-PAGE and silver staining of garvicin Q samples were performed according to a previously published protocol [63]. For zymogram analysis the silver-stained gel was washed for 3 h in HPLC-grade water and then placed on a BHI agar plate containing embedded *L. innocua* LMG2785 at an OD_600_ of ~ 0.002. The agar plate was incubated overnight at 37 °C and imaged with a digital camera.

### Adsorption assay

Adsorption of garvicin Q to *L. innocua* LMG2785, *L. lactis* IL1403, *L. monocytogenes* EGDe, *L. monocytogenes* EGY2, *L. petauri* B1726 and *C.* *glutamicum* ATCC13032 cells was analysed as described previously [65] with modifications. Overnight cultures were washed three times with phosphate-buffered saline. Bacteria were then resuspended at an OD_600_ of 3 in 5 mM sodium phosphate buffer (pH 6.5) containing HIC purified garvicin Q. Where indicated, a cocktail of protease inhibitors (cOmplete™ Protease Inhibitor Cocktail, Sigma) with 1 mM EDTA was added to assays to exclude proteolytic degradation of garvicin Q. The peptide-cell mixture was incubated for 1 h at 30 (*Lactococcus* spp. and *C. glutamicum*) or 37 °C (*Listeria* spp.). Then, bacteria were precipitated by centrifugation and bacteriocin activity was measured in the adsorption supernatants (ASN) using a growth inhibition assay with *L. lactis* IL1403 as described above. The percentage of adsorbed garvicin Q was calculated according to following formula:

$$\mathrm{adsorption} \left[\%\right]=100-\left(\frac{\mathrm{bacteriocin\, activity\, in\, ASN}}{\mathrm{original\, bacteriocin \,activity}}\times 100\right)$$

### Time-kill assay

Time-kill assays were performed with *L. innocua* LMG2785 and *L. lactis* IL1403 [66]. BHI or GM17 were inoculated with the respective strains to an initial OD_600_ of 0.5. HIC purified garvicin Q (16-fold diluted), chloramphenicol (10 µg ml^−1^), nisin (100 µg ml^−1^) or H_2_O was added to the cultures. After 0, 2, 4, and 6 h tenfold serial dilutions of the cultures were plated on BHI or GM17 agar plates. The agar plates were incubated overnight at 30 or 37 °C and colony forming units (cfu) were determined by counting single colonies in appropriate dilutions.

## Results

### *Lactococcus* sp. B1726 produces a protease sensitive, amphiphilic bacteriocin

**Fig. 1:**
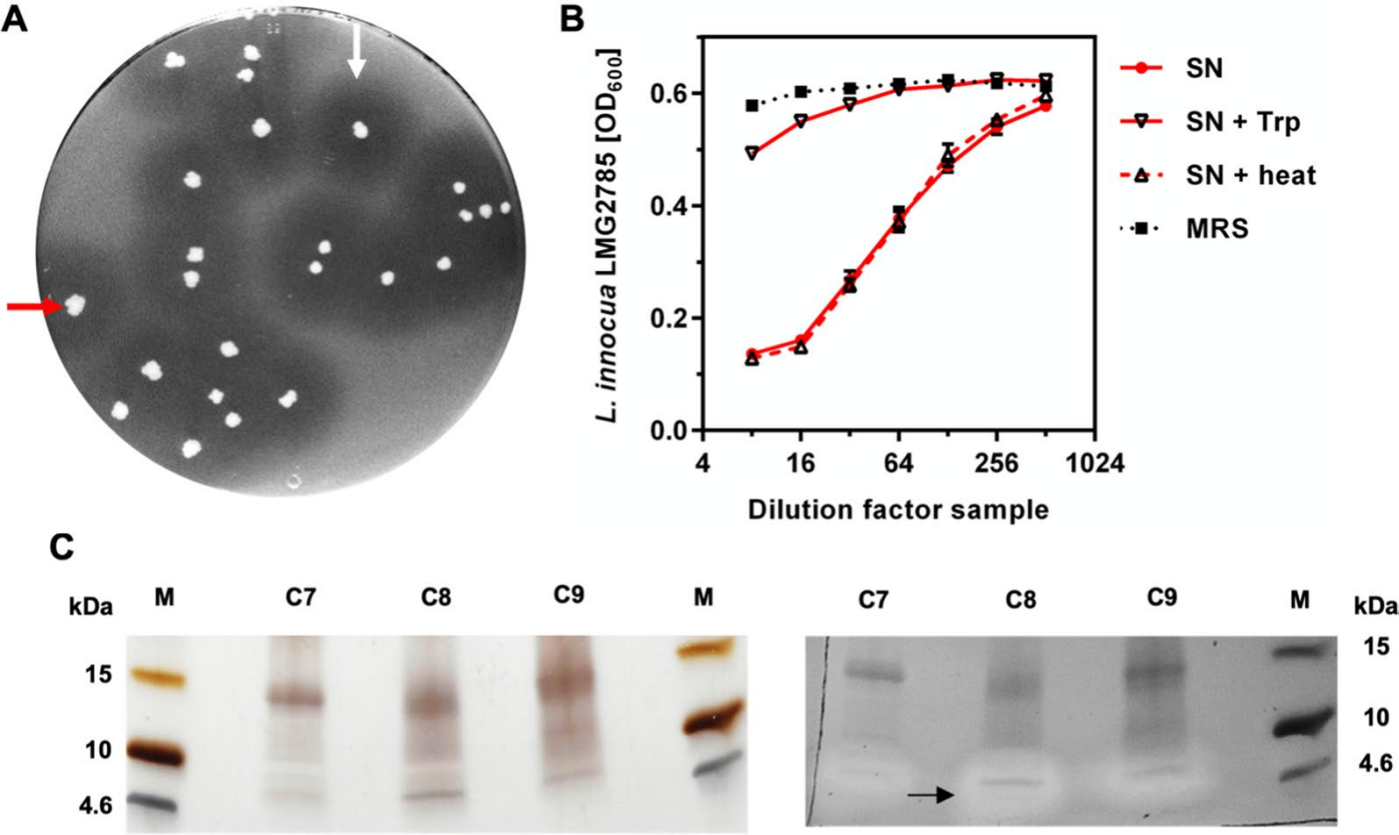
*Lactococcus* sp. B1726 produces a heat-stable and protease sensitive bacteriocin. **A** Overlay agar plates of *Lactococcus* sp. B1726 colonies with *L. innocua* LMG2785 as indicator strain. The red arrow marks a single colony *of Lactococcus* sp. B1726 and the white arrow highlights a zone of inhibition surrounding *Lactococcus* sp. B1726. **B** Growth inhibition assay of serial twofold dilutions of supernatants (SN) of *Lactococcus* sp. B1726. Where indicated SN were treated with trypsin (Trp) or incubated at 80 °C for 15 min (heat). MRS medium served as negative control. *L. innocua* LMG2785 was used as indicator strain. Values are mean ± standard deviation of n = 3 independent cultures. **C** Silver stained SDS-gel (left) and zymogram (right) of RPC fractions (C7-C9) of *Lactococcus* sp. B1726 SN. The black arrow indicates the antimicrobial active peptide. M = Spektra^™^ Low range ladder

*Lactococcus* sp. B1726 was initially isolated from fermented balsam pear during a screening for potential bacteriocin producers with activity against pathogenic *L. garvieae* [67]. To corroborate these results, we tested growth inhibition of *Listeria innocua* LMG2785 by *Lactococcus* sp. B1726 colonies on overlay agar plates (Fig. 1A). A clear zone of inhibition surrounding single colonies of *Lactococcus* sp. B1726 was observed indicating the production of a compound with activity against *L. innocua* LMG2785. This result was confirmed in growth inhibition assays in a microtiter plate format using serial dilutions of culture SNs of *Lactococcus* sp. B1726 (Fig. 1B). While untreated *Lactococcus* sp. B1726 SNs showed potent inhibition of growth of *L. innocua* LMG2785, treatment of SNs with trypsin efficiently abolished this activity. By contrast, heat treatment (80 °C, 15 min) of SNs had no effect.

To test the cationic and hydrophobic character of the *Lactococcus* sp. B1726 antimicrobial protein or peptide, the compound was concentrated by ammonium sulphate (AS) precipitation, cation exchange chromatography (CIEX) and reverse-phase chromatography (RPC; Additional file 1: Figure S2). Zymogram analysis of RPC elution fraction revealed a single band of a molecular mass of approx. 5 kDa with antimicrobial activity against *L. innocua* LMG2785 (Fig. 1C). These findings suggest that *Lactococcus* sp. B1726 secretes a heat-stable and protease-sensitive antimicrobial protein or peptide and are consistent with characteristics of low molecular weight bacteriocins.

### Genome analysis and identification of the putative *gar* operon of *Lactococcus* sp. B1726

The genome of *Lactococcus* sp. B1726 was sequenced using Illumina and ONT and assembled to a single circular contig. The genome has a size of 2,106,259 bp and a GC content of 38%, which is in line with previously sequenced *L. garvieae* and *L. petauri* genomes [14, 68, 69]. Initial whole genome sequence alignment proposed that the isolate belongs to the species *L. garvieae* as the closest relative was *L. garvieae* PAQ102015-99 with an average nucleotide identity (ANI) of 99.94% and a coverage of 97.39% (Additional file 1: Table S3). However, more recent ANI analyses suggest that *L. garvieae* PAQ102015-99 should be reclassified to the species *Lactococcus petauri* [6, 69], which was first isolated from an abscess of a sugar glider (*Petaurus breviceps*) and more recently was also described to cause lactocococcis in fish [6, 69]. According to ANI, the most closely related type strain of our isolate was *L. petauri* 159,469 (98.55%) whereas the *L. garvieae* type-strain ATCC 49,156 showed lower ANI (93.30%) (Additional file 1: Table S3). Consequently, we classified our isolate B1726 the species *Lactococcus petauri*.

We searched the annotated *L. petauri* B1726 genome for putative virulence factors and genes described to be essential for survival of other pathogenic *L. garvieae* and *L.* *petauri* strains in the host [6, 8, 70–72]. We identified various genes coding for adhesins, stress response proteins, and proteins involved in host-adapted metabolism (Additional file 1: Table S4). Moreover, *L. petauri* B1726 harbours genes involved in modification of the cell envelope including phosphoglucomutase and D-alanine-D-alanyl carrier protein ligase which may play a role in immune evasion. Of note, a gene coding for a putative hemolysin A was identified. Indeed *L. petauri* B1726 showed clear α-haemolytic activity on blood agar plates after incubation at 37 °C (Additional file 1: Figure S5). Interestingly, no *lacG* homologue for phospho-β-galactosidase was identified in the genome of *L. petauri* B1726. This indicates that the strain is not able to utilize lactose as a carbon source.

**Fig. 2:**
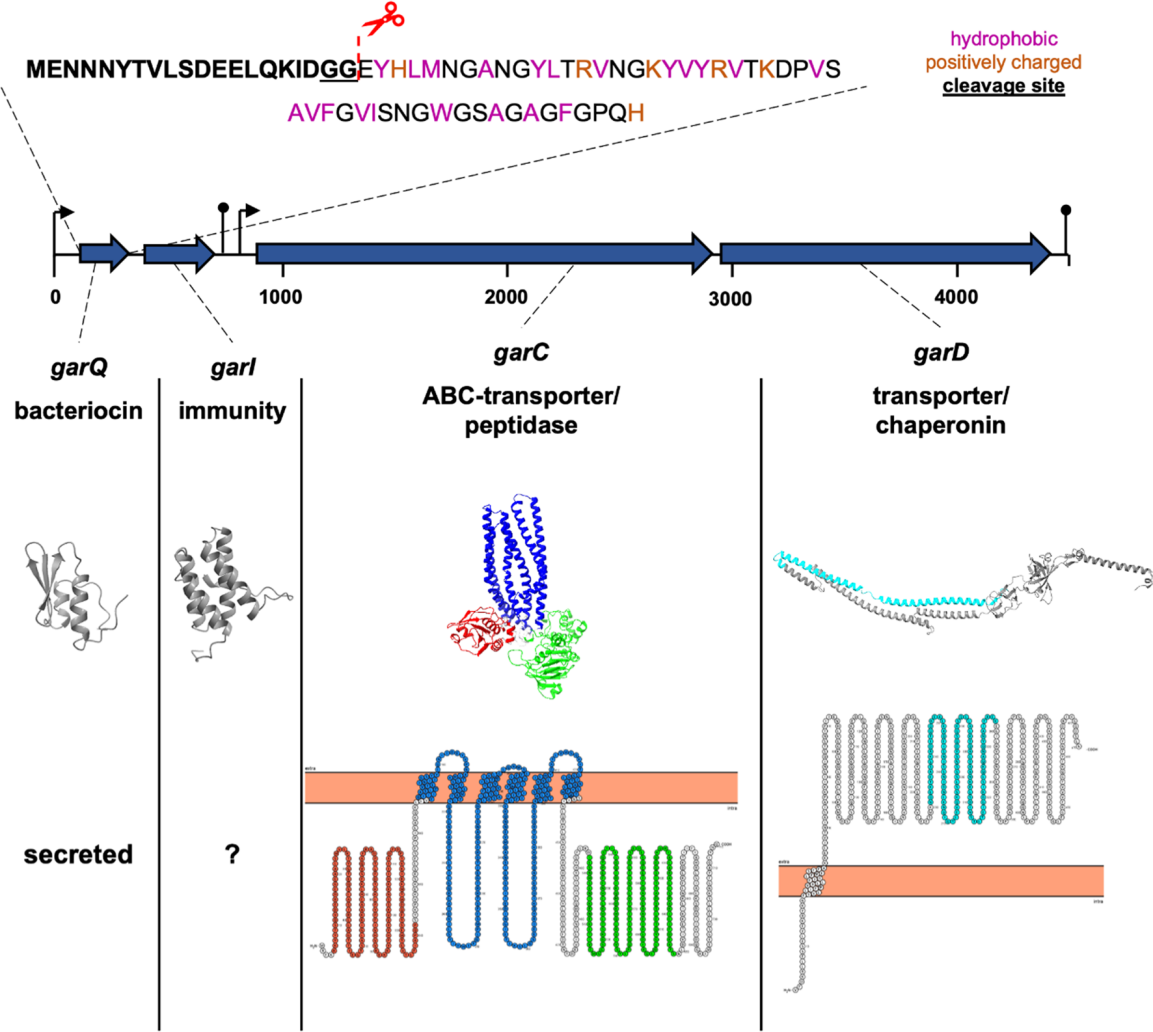
Putative bacteriocin gene cluster of *L. petauri* B1726 for production of garvicin Q. The cluster consists of four genes: *garQ* for the pre-peptide, *garI* for an immunity protein, *garC* and *garD* for the transporter. The cluster contains two predicted promoters upstream of *garQ* and *garC* (indicated by arrows) and two Rho-independent transcription terminators downstream of *gari* and *garD* (indicated by lollies). Pre-garvicin Q consists of a N-terminal signal sequence (bold) with a conserved double glycine cleavage site (underlined). Pre-garvicin Q is likely processed by GarC during secretion. GarI shows similarity to an enterocin A immunity protein (PF08951). GarC contains an intracellular C39 peptidase domain (red, PF03412), an ABC-transporter transmembrane part (blue, PF00664) and an intracellular ATP-binding domain (green, PF00005). GarD contains an extracellular HlyD -typical motif (light blue, PF13437)

To identify potential bacteriocin gene clusters, the genome of *L. petauri* B1726 was searched with the online tools BAGEL4 and antiSMASH. Both tools identified one region of interest consisting of four genes that were designated as *garQICD* (Fig. 2A, Additional file 1: Figure S6). The first gene, *garQ* (*lgb_01492*), is predicted to encode a peptide precursor identical to the previously described garvicin Q [23]. Similarly, *garI* (*lgb_01493*), encodes a putative bacteriocin immunity protein with similarity to an enterocin A immunity protein (PF08951). The genes *garCD* (*lgb_01494* and *lbg_01495*) encode for an ABC-transporter with a C-terminal C39 peptidase domain (PF00005, PF00664 and PF03412), and an accessory secretion protein with a HlyD typical motif (PF13437). Of these four genes, *garQ*, *garI* and *garC* are almost identical to the genes *garQ*, *garI* and *garT* of *L. garvieae* BCC 43,578 (GenBank Accession Number JN605800) previously shown to be associated with garvicin Q production [23]. In silico analysis using BPROM predicted promoters upstream of *garQ* and *garC* and putative rho-independent transcription terminators were identified downstream of *garI* and *garD*. This suggests that the four genes are organised in two bicistronic operons with transcripts for *garQI* and *garCD*. A similar prediction was made for the garvicin A gene cluster of *L. garvieae* 21,881 [25].

The deduced amino acid sequences of GarC and GarD have a high sequence similarity to LgnC (97.62%) and LgnD (96.21%), i.e., the garvicin A transporter of *L. garvieae* (UniProtKB H2B2W2 and H2B2W2). GarC and GarD also have a similar amino acid sequence to LcnC (73.01%) and LcnD (56.96%), i.e. the lactococcin A transporter of *L. lactis* subsp. *lactis* (UniProtKB Q00564 and P0A3G5). Moreover, the N-terminal signal sequences of garvicin Q, garvicin A and lactococcin A share a conserved motif including a double glycine required for cleavage of the signal sequence by the transporter [26] (Fig. 2). In silico prediction of GarD membrane topology showed that it consists of a short N-terminal intracellular part and a long α-helical extracellular part (Fig. 2) and shares similarity with HlyD and LcnD 27. Altogether, in silico analyses indicated that *L.* *petauri* B1726 possesses all components required for the synthesis, immunity, and secretion of the bacteriocin garvicin Q.

### Optimization of cultivation conditions for garvicin Q production

**Fig. 3:**
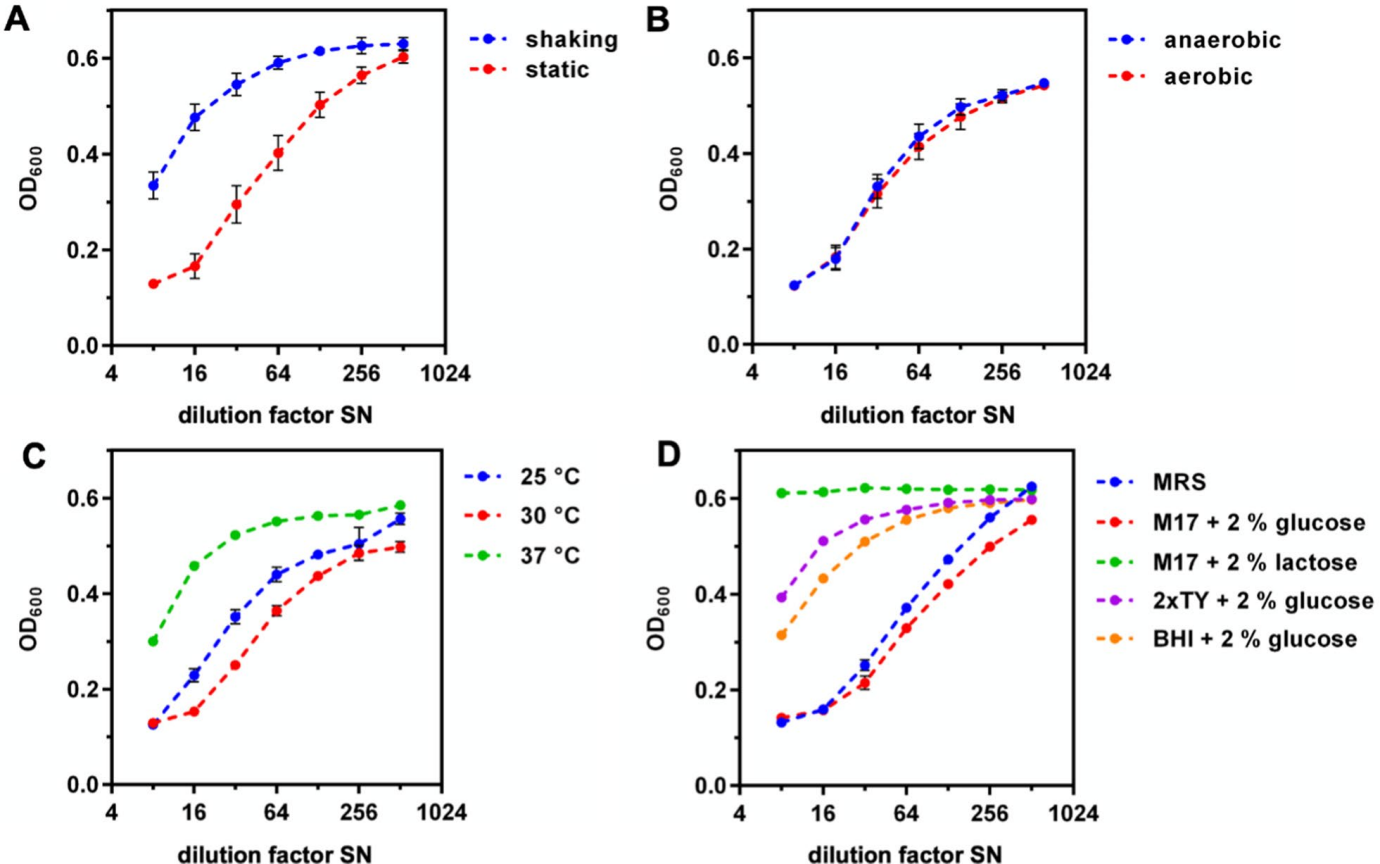
Antimicrobial activity in SN of *L. petauri* B1726 cultivated under different conditions. *L. petauri* B1726 was grown in MRS medium overnight at 30 °C under static conditions or with shaking A, statically under aerobic and anaerobic conditions **B**, statically at 25, 30 or 37 °C **C** or in the indicated media statically at 30 °C **D**. Activity was tested by incubating the indicator strain *L. innocua* LMG2785 with serial twofold dilutions of supernatants (SN). Values are mean ± standard deviation of SN of n = 3 independent cultures

To increase the yield of garvicin Q for further analyses, the impact of different cultivation conditions and media on the production of garvicin Q by *L. petauri* B1726 was tested (Additional file 1: Table S7) and antimicrobial activity (BU/ml) was determined after o/N cultivation. In MRS medium, SNs obtained after static cultivation showed higher activities compared to SN of shaken *L. petauri* B1726 cultures (Fig. 3A), which may be associated to reduced growth and more alkaline pH in shaken cultures (Additional file 1: Table S7). To exclude a detrimental effect of oxygen on production and stability of garvicin Q, *L. petauri* B1726 was cultivated statically under aerobic and anaerobic conditions. No differences in final optical densities or activity in SNs were observed between aerobic and anaerobic conditions (Fig. 3B), indicating that strictly anaerobic conditions are not required for garvicin Q production but shaking leads to lower biomass yield and reduced activity in SN. The cultivation temperature affected the garvicin Q production by *L. petauri* B1726 (Fig. 3C).

Interestingly, growth of *L. petauri* B1726 and pH of the cultures were comparable at 25, 30, and 37 °C (Additional file 1: Table S7) but highest activities were obtained with cultures grown at 30 °C (Fig. 3C). Also, the impact of different media on antimicrobial activity in SNs of *L. petauri* B1726 was investigated in static cultures (Fig. 3D, Additional file 1: Table S7). Very poor growth and activity was observed in M17 + 2% lactose, indicating that *L. petauri* B1726 is unable to efficiently consume lactose. This fits nicely to the lack of a *lacG* homologue in the genome of *L. petauri* B1726. Final optical densities (OD_600_) of *L. petauri* B1726 in 2xTY + 2% glucose, BHI + 2% glucose, and MRS medium were comparable. However, activities in TY and BHI were marginal compared to MRS. Cultivation of *L. petauri* B1726 in M17 + 2% glucose (30 °C, static) resulted in the highest final OD_600_ and activities (OD_600_ 2.8, 1280 BU/ml).

**Fig. 4:**
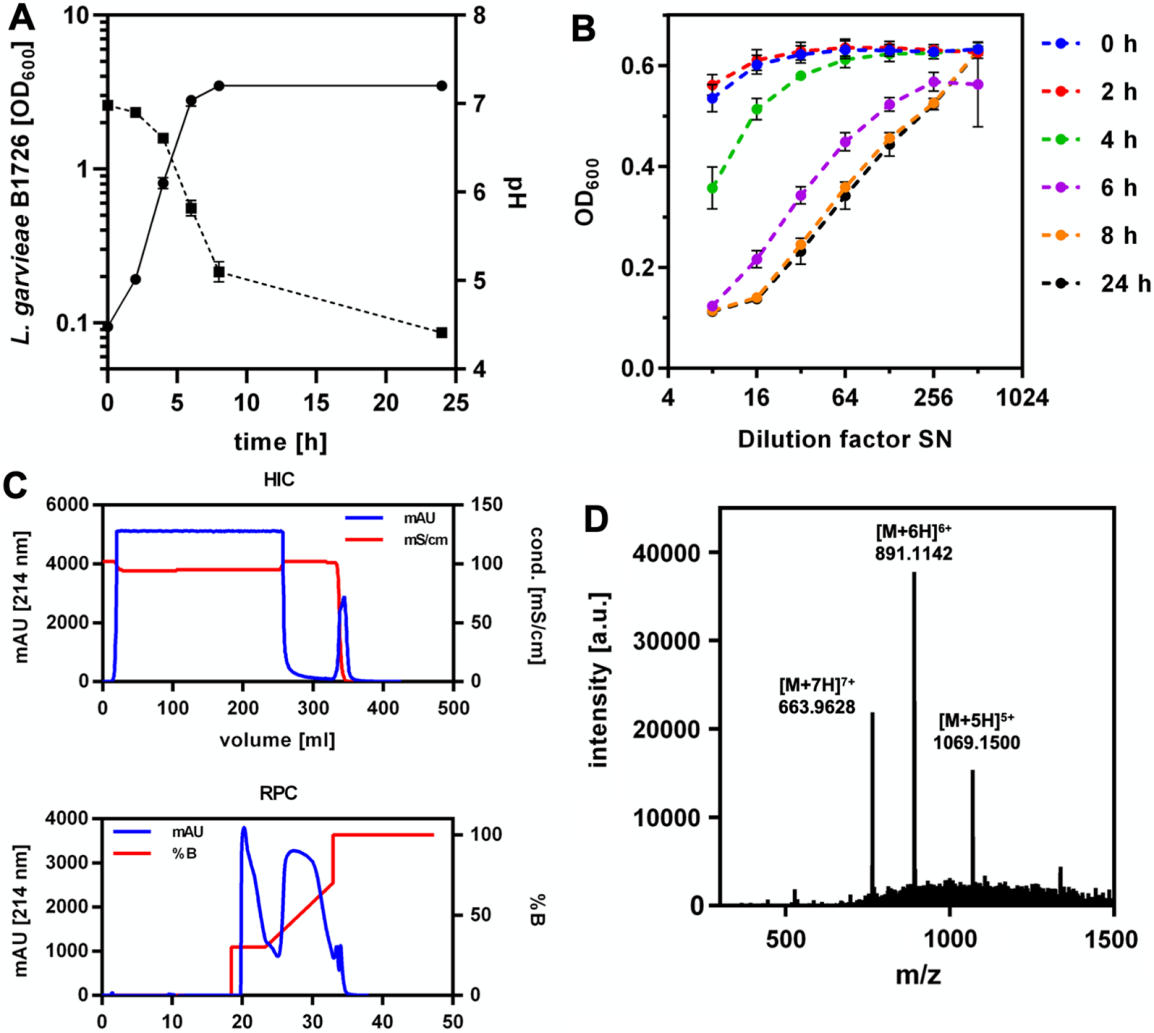
Kinetics of production, purification, and mass spectrometry analysis of garvicin Q of *L. petauri* B1726. **A** Optical density (OD_600_, solid line) and pH (dashed line) of *L. petauri* B1726 cultures grown in M17 medium supplemented with 2% glucose. The cells were incubated statically in 50 ml Schott glass bottles at 30 °C. **B** Antimicrobial activity in SN from (**A**) harvested at indicated time points. Activity was tested by incubating the indicator strain *L. innocua* LMG2785 with serial two-fold dilutions of SN. Values in (**A**) and (**B**) are mean ± standard deviation of n = 3 independent cultures. **C** Hydrophobic interaction chromatography (upper panel) and subsequent reverse-phase chromatography (lower panel) to purify garvicin Q from *L.* *petauri* B1726 SN after cultivation for 24 h in M17 medium supplemented with 2% glucose **A**. **D** LC–MS analysis of combined RPC elution fraction harbouring antimicrobial activity. The peaks with mass to charge ratios (m/z) of 1069.1500, 891.1142 and 763.9628 correspond to garvicin Q with five, six or seven positive charges, respectively

Since M17 + 2% glucose performed best under the tested conditions, this medium and static cultivation at 30 °C were used for further experiments. To determine the best timepoint for collection of SNs, OD_600_, antimicrobial activity and pH were measured during cultivation of *L. petauri* B1726 (Fig. 4A, B). Under these conditions, the growth rate of *L. petauri* B1726 in exponential phase was 0.67 h^−1^ and cultures entered stationary phase after 8 h at an OD_600_ of 2.8. The pH of the cultures continuously decreased during growth and reached a value of 4.4 after 24 h of cultivation. Antimicrobial activity was detected first after 4 h of cultivation (OD_600_ approx. 0.8, pH 6.6, 160 BU/ml), reached a maximum after 8 h (OD_600_ of 3.5, pH 5.1, 1280 BU/ml), and remained stable until the end of the experiment. After 24 h, SNs were collected and subjected to HIC and subsequent RPC (Fig. 4C). Here, AS precipitation and CIEX were replaced by HIC, since it was observed that AS precipitation results in problems with solubility of the hydrophobic garvicin Q (Additional file 1: Table S8). Combined RPC elution fractions containing antimicrobial activity were analysed by mass spectrometry (Fig. 4D, Additional file 1: Figure S9). Three distinct peaks with a mass/charge ratio (m/z) of 1069.1500, 891.1142 and 763.9628 were observed corresponding to the five-, six and seven-fold charged ions of garvicin Q, respectively (Fig. 4C and Additional file 1: Figure S9). The experimentally determined monoisotopic mass of 5346.72 Da fits to the calculated monoisotopic mass of garvicin Q (5340.63 Da). MS of the samples after tryptic digestion confirmed the identity of garvicin Q (Additional File 2).

**Fig. 5:**
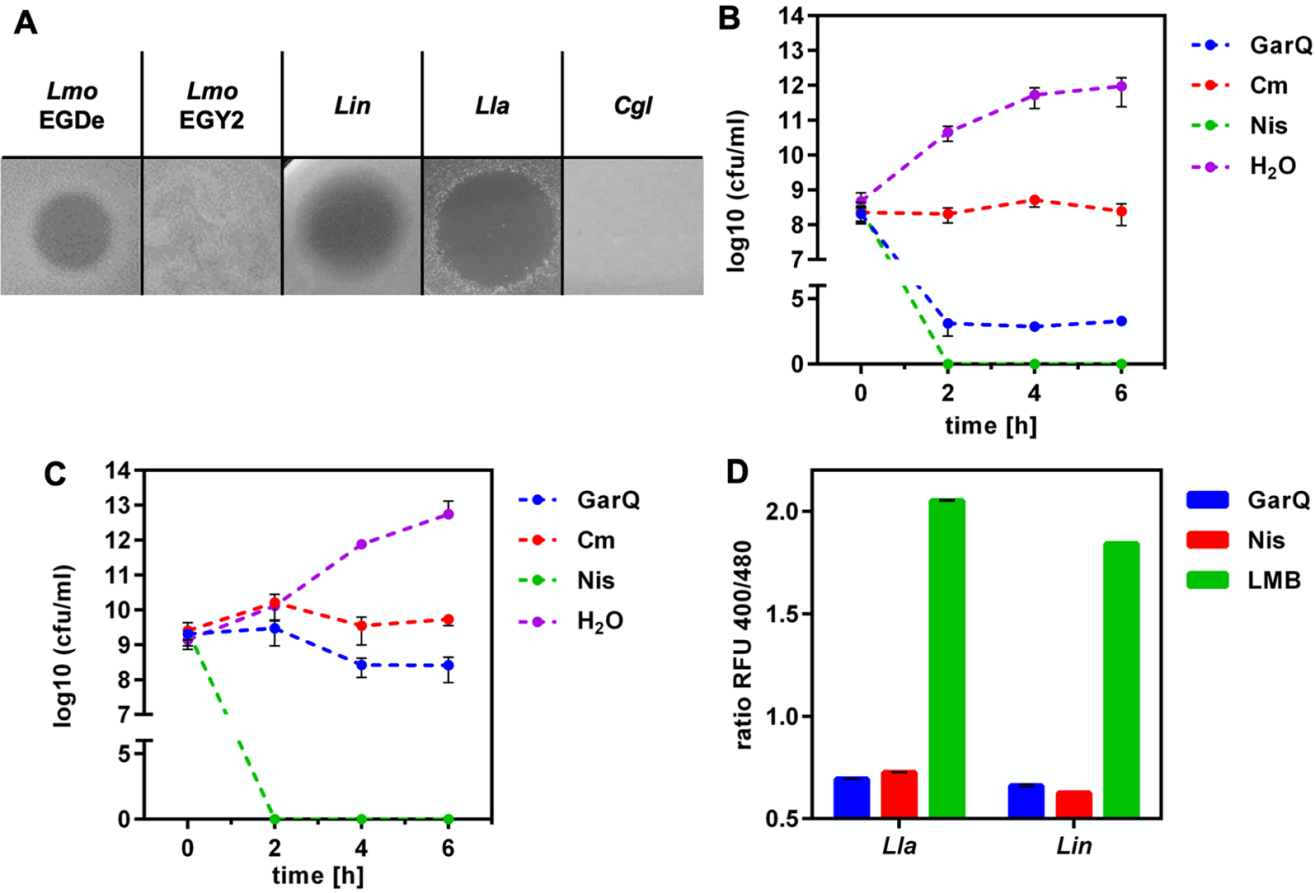
Mode of action of garvicin Q. **A** Sensitivity of *L. monocytogenes* EGDe, *L. monocytogenes* EGY2, *L.* *innocua* LMG2785, *L.* *lactis* IL1403 and *C.* *glutamicum* ATCC13032 towards purified garvicin Q. **B** and **C** Viable cell count (log cfu/ml) of *L.* *lactis* IL1403 **B** and *L.* *innocua* LMG2785 **C** after treatment with garvicin Q (GarQ). As controls, bacteria were treated with nisin (Nis; bacteriolytic), chloramphenicol (Cm; bacteriostatic) or water (H_2_O; negative control). **D** Fluorescence intensity ration with emission at 520 nm after excitation at 400 and 480 nm (ratio RFU 400/480) biosensors *L. innocua* LMG2785/pNZ-pHin2^*Lm*^ (*Lin*) and *L. lactis* IL1403/pNZ-pHin2^*Lm*^ (*Lla*) following treatment with a garvicin Q RPC preparation (GarQ), and nisin (6.25 µg/ml; Nis) compared to unreated controls (LMB). All values are mean ± standard deviation of n = 3 independent cultures

Previous reports have shown that garvicin Q from *L. garvieae* BCC 43,578 exhibits a broad antimicrobial spectrum and requires the IIC and IID subunits of the PTS^Man^ as receptors at target cells for activity [23, 73]. To confirm the PTS^Man^-dependent antimicrobial activity of garvicin Q, we tested susceptibility of different bacteria either harbouring a group I PTS^Man^ (*L. lactis* IL1403, *L. innocua* LMG2785, *L. monocytogenes* EGDe) or not (*L. monocytogenes* EGY2 and *C. glutamicum* ATCC13032) to purified garvicin Q (Fig. 5A). Growth of *L. monocytogenes* EGDe, *L. innocua* LMG2785, and *Lactococcus lactis* IL1403 were inhibited by purified garvicin Q. By contrasts *L.* *monocytogenes* EGY2, which harbours a deletion in the extracellular γ-region of the PTS^Man^ IID subunit rendering the strain resistant to the class II bacteriocins mesentericin and pediocin [36, 63], and *C. glutamicum* ATCC13032 that does not possess a PTS^Man^ were completely resistant. Interestingly, a potential interaction of garvicin Q with the IIC and IID subunits of PTS^Man^ could be predicted in silico (Additional file 1: Figure S10). The predicted garvicin Q–PTS^Man^ complex is similar compared to the Cryo-EM structure of the pediocin–PTS^Man^ complex ([74] and Additional file 1: Fig. 11). PTS^Man^ residues previously identified to be involved in interaction with garvicin Q [73] and pediocin [74] were located in close vicinity to garvicin Q. This suggests a similar mode of action of pediocin and garvicin Q. In summary, these findings support the hypothesis of Tymoszewska et al. [73] that the PTS^Man^ serves as receptor for garvicin Q.

**Fig. 6:**
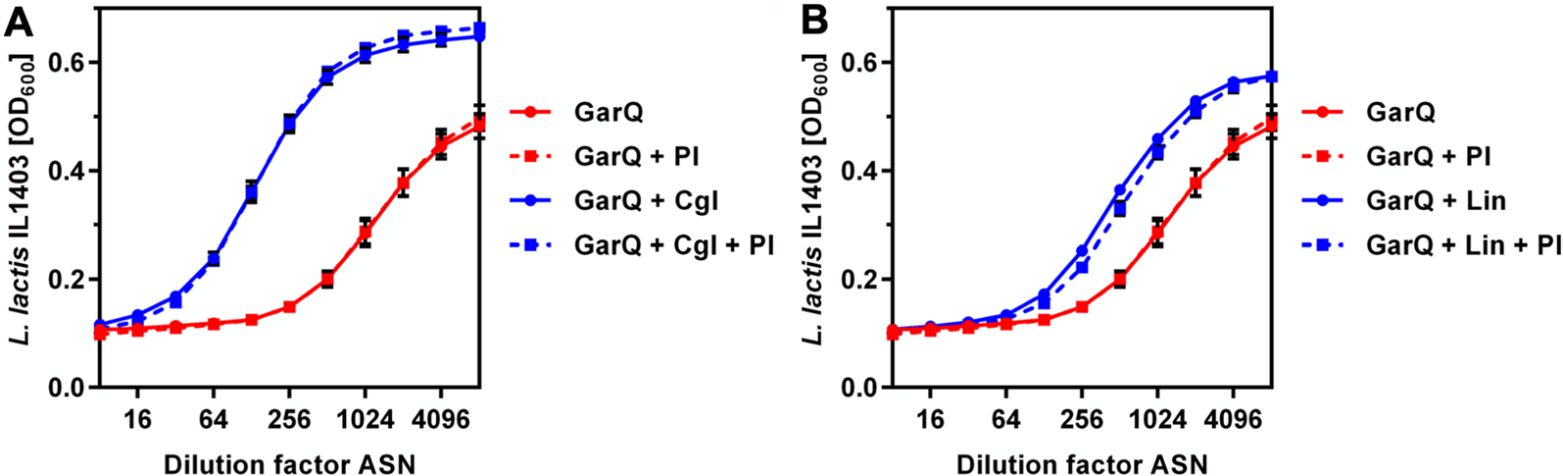
Garvicin Q adsorption assay in the presence of protease inhibitors. Activity of garvicin Q (GarQ, red lines) in following incubation with *C. glutamicum* ATCC13032 (**A**; Cgl, blue lines) or *L. innocua* LMG2785 (**B**; Lin, blue lines) was tested with (solid lines) or without (broken lines) presence of a protease inhibitor cocktail with 1 mM EDTA (PI). Activity was tested by measuring optical density (OD600) after incubation of the indicator strain *L. lactis* IL1403 with serial twofold dilutions of supernatants of adsorption assays (ASN) with the indicated treatment combinations. Values are mean ± standard deviation of n = 3 independent assays

We then investigated the binding of garvicin Q to the same set of bacteria plus the native producer *L. petauri* B1726 by adsorption assays with purified garvicin Q (Table 2). Activity in garvicin Q preparations was strongly reduced (> 80%) after incubation with cells of all strains except *L. petauri* B1726, i.e., the natural producer. This indicates that garvicin Q binds to a wider range of bacteria, but antimicrobial activity requires the presence of a functional PTS^Man^. To rule out proteolytic degradation as the mechanism of reduced garvicin Q activity following incubation with bacterial biomass, we performed additional adsorption assays in the presence and absence of protease inhibitors with representatives of susceptible (*L. innocua* LMG2785) and resistant (*C. glutamicum* ATCC13032) bacteria. In both cases, garvicin activity was largely abolished following incubation with bacteria and addition of the protease inhibitor cocktail had no effect, suggesting that proteolytic degradation of garvicin Q is not responsible for the loss in activity (Fig. 6).

**Table 2:** Adsorption of garvicin Q to different Gram-positive bacteria with or without a group I PTS^Man^

Strain	Adsorption [%]	Group I PTS^Man^
*L. lactis* subsp. *lactis* IL1403	88	Yes
*L. innocua* LMG2785	88	Yes
*L. monocytogenes* EGDe	94	Yes
*L. monocytogenes* EGY2	88	Truncated
*L. petauri* B1726	50	No
*C. glutamicum* ATCC13032	94	No

To further investigate the mode of action of garvicin Q, viable cell counts of two bacteria targeted by garvicin Q (*L. innocua* LMG2785 and *L. lactis* IL1403) were determined after treatment with this bacteriocin (Fig. 5B and C). Both target bacteria showed similar behaviour. Treatment with chloramphenicol had a clear bacteriostatic effect on both indicator bacteria and growth was observed in samples treated with water. Similar to the bactericidal bacteriocin nisin, treatment with garvicin Q led to a reduction in viable cell count over time suggesting a bactericidal mode of action of garvicin Q. This effect was more pronounced for *L. lactis* IL1403.

To identify the underlying mechanism of the bactericidal effect of garvicin Q, we conducted assays with previously described *L. innocua* [34] and newly generated *L. lactis* pHluorin2 biosensor bacteria (Additional file 1: Figure S12). These sensors allow detection of intracellular pH-shift due to disruption of membrane integrity by e.g. bacteriocins [12, 63, 75, 76]. Both sensor strains clearly showed perturbed intracellular pH following treatment with nisin or garvicin Q as indicated by a drop in fluorescence intensity ratios compared to the untreated controls (Fig. 5D). This points towards a membrane-damaging mechanism as described for e.g. pediocin [74, 77].

### Heterologous expression of *garQICD* results in synthesis of garvicin Q

To conclusively demonstrate that the genes of the *gar* locus are responsible for biosynthesis of garvicin Q, they were expressed in *C. glutamicum*. This organism was selected as a host as it was shown to be resistant to garvicin Q, produces no own bacteriocin and was recently successfully engineered for production of the bacteriocins pediocin PA1 and (pre)nisin [63, 76]. The sequences of *garQ*, *garI*, *garC*, and *garD* were optimized for codon usage of *C. glutamicum*, each equipped with the strong ribosome binding site of the *tuf* gene of *C. glutamicum* and cloned into the vector pPBEx2 under control of IPTG-inducible *tac* promoter. The obtained plasmid pPBEx2-*garQICD*^*Cgl*^ (Additional file 1: Figure S13A) was introduced into *C. glutamicum* CR099. First experiments with a standard protocol for induction only in the main culture did not yield detectable activities in SNs of *C. glutamicum* CR099/pPBEx2-*garQICD*^*Cgl*^ (Additional file 1: Figure S13B). We thus turned to a protocol previously successfully used for production of prenisin [76] that includes induction of precultures using 0.05 mM IPTG and main cultures using 0.2 mM IPTG (Additional file 1: S13C). Activity assays on these SNs indicated presence of an antimicrobial (Additional file 1: figure S12B). This activity was observed mainly in the early phases of cultivation, i.e. after 4 h, and largely reduced at the end (24 h).

**Fig. 7:**
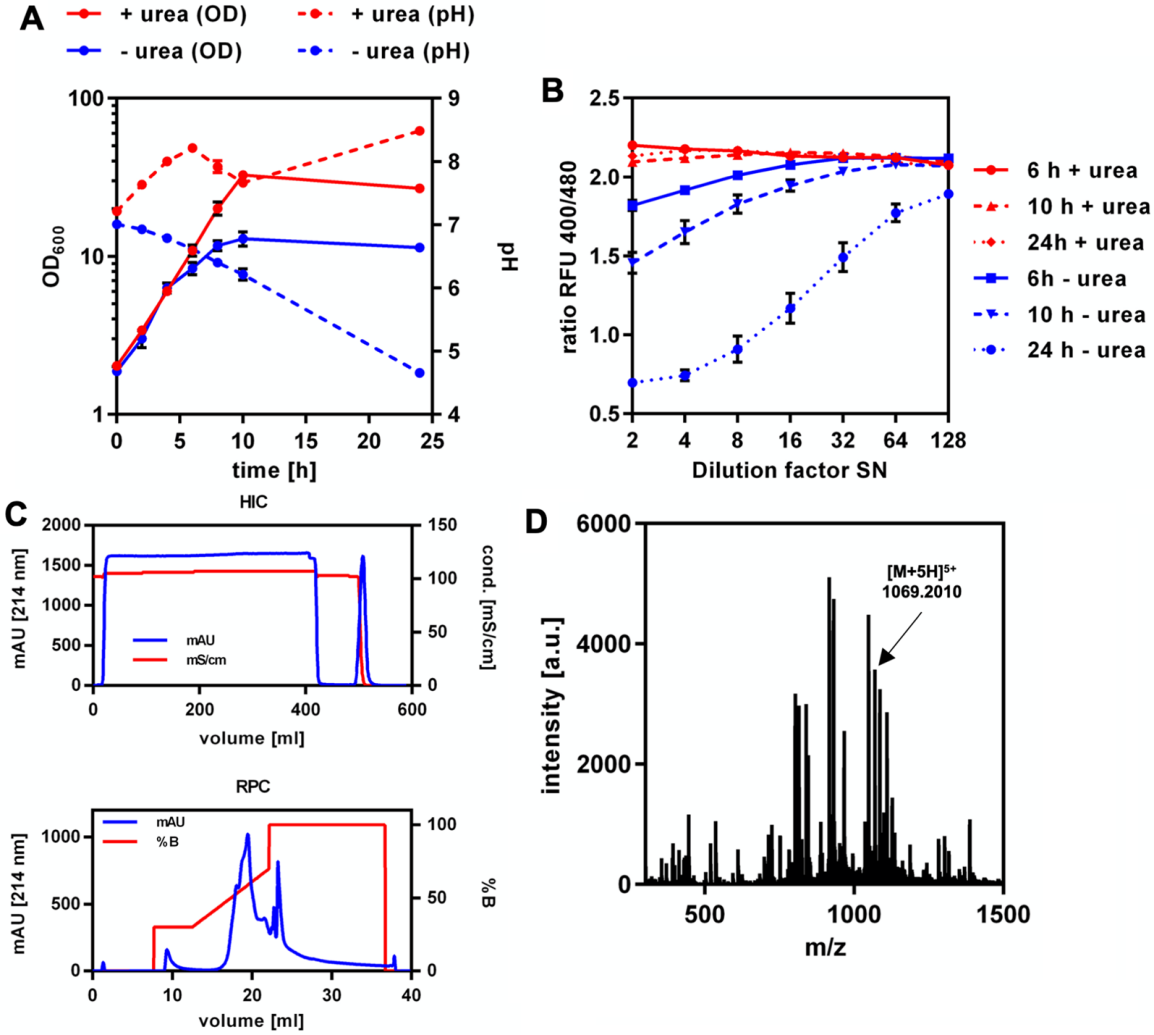
Heterologous expression of *garQICDCgl* in *C. glutamicum*. **A** Optical density (OD600; solid lines) and pH (broken lines) in supernatants of *C. glutamicum* CR099/pPBEx2-*garQICDCgl* cultivated in CGXII + 2% glucose with (red lines) or without (blue lines) urea. **B** antimicrobial activity in SNs of these cultivations sampled at the indicated timepoints. Activity was assayed using *L. lactis* IL1403/pNZ-pHin2*Lm* as indicator. Values are ratios of fluorescence intensity at 520 nm after excitation at 400 and 480 nm (ratio RFU 400/480) and are mean of n = 3 independent experiments (supernatants of independent cultivations). **C** Purification of garvicin Q from SN of *C. glutamicum* CR099/pPBEx2-*garQICDCgl* after cultivation for 24 h in CGXII medium + 2% glucose without urea by hydrophobic interaction chromatography (HIC; upper panel) and subsequent reverse-phase chromatography (RPC; lower panel). **D** LC–MS analysis of pooled RPC elution fractions harbouring antimicrobial activity. The peak with a mass to charge ratio (m/z) of 1069.2010 corresponds to garvicin Q with five positive charges

To further improve heterologous production of garvicin Q, further cultivations of *C. glutamicum* CR099/pPBEx2-*garQICD*^*Cgl*^ were performed in defined CGXII medium (Fig. 7). Surprisingly, no activity was detected in SNs under these conditions despite growth to high optical densities (Fig. 7B). The pH steadily increased over the course of the fermentation (Fig. 7A). As highest garvicin Q levels in SNs of the natural producer *L. petauri* B1726 are observed in late exponential to stationary phase when cultures had a pH of 4–5 [78], we hypothesized that acidic pH during cultivation might increase garvicin Q production. Thus, we performed additional experiments in CGXII medium without urea to allow acidification. Indeed, lack of urea resulted in a continuous acidification of culture during growth (Fig. 7A). Moreover, significant activity was detected in *C. glutamicum* CR099/pPBEx2-*garQICD*^*Cgl*^ SNs at all timepoints during the cultivation on CGXII medium without urea (Fig. 7B) despite significantly lower final OD_600_. Moreover, activity steadily increased over the course of the experiment and reached levels significantly higher than in the initial experiments on 2TY medium with glucose (Additional file 1: Figure S13). To confirm the identity and correct processing of the heterologous produced garvicin Q, the recombinant peptide was purified by HIC and RPC (Fig. 7C) as described for GarQ of the natural producer *L. petauri* B1726. LC–MS analysis of the RPC fraction containing antimicrobial activity yielded a signal with m/z = 1069.2010 corresponding to the five-fold charged ion of garvicin Q (Fig. 7D and Additional file 1: Figure S14). MS of the samples after tryptic digestion confirmed the identity of recombinant garvicin Q (Additional File 3).

## Discussion

The class IId bacteriocin garvicin Q was previously shown to be produced by *L. garvieae* [23]. It is active against a wide spectrum of target bacteria including *Lactococcus* spp., *Lactobacillus* spp., *Leuconostoc* spp., *Pediococcus* spp. *Enterococcus* spp. and *Listeria* spp. [23, 73]. Moreover, it does not show cytotoxicity towards Vero and HepG2 cell lines, implicating the potential of garvicin Q for therapeutic applications [23].

Our data shows that *L. petauri* B1726 produces a heat-stable, protease sensitive, amphiphilic bacteriocin with a molecular mass of ~ 5 kDa. To unravel the genetic traits associated with production of this antimicrobial protein/peptide, we sequenced, annotated, and analysed the genome of *L. petauri* B1726. It contains several genes that encode for virulence factors and proteins for survival in the host described for pathogenic *L. garvieae* and *L. petauri* strains [6, 8, 70–72]. Also, no *lacG* homologues required for lactose utilization were found in the *L. petauri* B1726 genome and the strain showed poor growth on M17 medium containing lactose. Inability to utilize lactose is another feature associated with pathogenic *L. garvieae* isolates [79]. Collectively, these findings suggest that *L.* *petauri* B1726 is a strain with pathogenic potential for fish and humans. This poses a serious problem for production of garvicin Q using its native producer and the European Food Safety Agency recently raised safety concerns over use of *L. garvieae* in food and feed [80].

Bioinformatic analyses revealed a complete operon for garvicin Q biosynthesis, which was only partially described before [23], and the peptide was identified in precipitated and purified SN proteins by mass spectrometry. The *gar* operon consists of four genes *garQ* (coding for the pre-peptide), *garI* (for an immunity protein), *garC* (ABC-transporter) and *garD* (additional protein presumably involved in secretion). The predicted immunity protein GarI contains an enterocin A immunity domain, and the predicted structure is similar to the antiparallel four alpha-helical bundle of enterocin A immunity protein [81]. The pediocin immunity protein is known to interact with the C-terminal domain of pediocin [82] and it is hypothesized that this somehow blocks the pediocin-induced formation of a channel in the PTS^Man^ from the cytoplasmic side [74]. Since garvicin Q also targets the PTS^Man^ [73], GarI might fulfil a similar role. This is supported by the fact that no N-terminal signal peptide could be identified in the GarI sequence suggesting it is localized intracellularly.

Similar to pediocin, the ABC-transporter GarC may not only be involved in secretion but also in processing of the garvicin Q pre-peptide after the double glycine motif at the end of the garvicin Q signal peptide, which is common among class II bacteriocins [83]. The role of the additional secretion protein GarD for garvicin Q production remains unclear. In *L. lactis* subsp. *lactis*, the GarD homolog LcnD was demonstrated to be essential for the secretion of lactococcin A and may stabilize the associated ABC-transporter LcnC [84]. Moreover, *lcnD* encodes two in frame proteins (a transmembrane protein and a cytosolic variant) in *L. lactis*. The truncated, cytosolic LcnD version is thought to act like a chaperone guiding lactococcin A to the ABC-transporter. Similarly, we identified a potential RBS and start codon within *garD*. Hence, it is possible that *garD* also encodes two proteins. It is noteworthy that GarD contains a HlyD-like motif. HlyD is a periplasmic adaptor protein and part of the type I secretion system of hemolysin in *E. coli*. HlyD monomers assemble in an α-helical nanotube structure that connect the inner and outer membrane transport proteins [27]. LcnD and GarD have a similar membrane topology as HlyD [85]. Based on these predictions it is tempting to speculate, that GarD also stabilizes GarC and builds an α-helical nanotube structure on top of GarC. Crystallization or further modelling approaches may help to prove this hypothesis.

We observed optimal garvicin Q production by *L. petauri* B1726 in static cultures. Shaking of *L. petauri* B1726 during cultivation decreased garvicin Q production and growth, presumably by increasing oxidative stress. The genome of *L. petauri* B1726 contains genes for enzymes involved in molecular oxygen consumption (NADH oxidase, *lgb_01366*) and detoxification of reactive oxygen species (superoxide dismutase, *lgb_00290*), however we could not identify a catalase gene. A lack of catalase may lead to H_2_O_2_ accumulation which would explain the reduced growth at shaking conditions. For *L. lactis* it has been demonstrated that accumulation of H_2_O_2_ reduces growth rate and recombinant expression of catalase alleviates oxidative stress [86, 87]. Temperature also affected garvicin Q production. Although the final OD_600_ and pH was comparable between *L. petauri* B1726 cultures incubated at 25, 30 and 37 °C, garvicin Q production was lowest at 37 °C. A similar temperature-dependent biosynthesis was also reported for the bacteriocin sakacin A of *Lactobacillus sakei* Lb706 with good production at 25 and 30 °C but not at 35 °C [88]. A peptide pheromone and a two-component system were shown to be involved in temperature-dependent regulation of biosynthesis of sakacin A and other class II bacteriocins [88, 89]. However, we could not find a similar regulatory system in close proximity to the *gar* operon of *L. petauri* B1726.

The PTS^Man^ of *L. lactis* IL1404 was shown to serve as a receptor for lactococcin A and its expression in a resistant *Lactobacillus sakei* strain installed sensitivity [90]. The subunits IIC and IID of group I PTS^Man^ were shown to bind garvicins A, B, C and Q and mutational analysis of *Lactococcus lactis* IL1403 and *L. garvieae* IBB3403 PTS^Man^ identified amino acids that might be involved in this interaction [73]. These amino acid residues are conserved in PTS^Man^ IIC and IID subunits of garvicin Q-sensitive bacteria and are localized in a transmembrane helix of the IIC subunit and in extracellular loops of the IID subunit [73]. It was proposed that the N-terminus of garvicin Q interacts with the residues located in the IID extracellular loops and that the α-helical C-terminus of garvicin Q interacts with the transmembrane residues of the IIC subunit. These interactions could then lead to structural rearrangements of the PTS^Man^ that creates a channel in the IIC permease resulting in disruption of membrane integrity [73]. Recently, the peptide-receptor complex could be resolved for pediocin and the *L. monocytogenes* PTS^Man^ [74] supporting this hypothesis. Our own structural modelling also predicts interaction of garvicin Q with the extracellular domain of the PTS^Man^ at the same site as pediocin [74] and in the vicinity of all the conserved amino acids required for activity of garvicin Q [73]. This further corroborates the hypothesis that the N-terminal β-sheet region of garvicin Q interacts with the extracellular part of the PTS^Man^ and its C-terminal α-helix cracks the PTS^Man^ to form a small hydrophilic pore with the PTS^Man^ subunits [73, 74].

Another interesting observation is that adsorption of garvicin Q to bacterial cells is not dependent on the presence of its receptor. Adsorption of garvicin Q to *C.* *glutamicum* ATCC13032 (no PTS^Man^) and to *L.* *monocytogenes* EGY2 (truncated IID of PTS^Man^) was in a similar range like adsorption to group I PTS^Man^ harbouring bacteria. Like other bacteriocins, garvicin Q is an amphiphilic peptide. Hence, unspecific interaction with negatively charged components of the Gram-positive envelope like phospholipids or teichoic acids could occur. Interestingly, adsorption to the native producer *L. petauri* B1726 was weaker compared to the other tested bacteria. This might point towards a modification of the *L. petauri* B1726 cell envelope to reduce binding of garvicin Q. This could be achieved by modifications of the cell envelope e.g. a positive surface charge and protection from cationic antimicrobials. In *Staphylococcus aureus*, such modifications are accomplished by sensing of cationic antimicrobials by the two-component system GraRS, lysinylation of phosphatidylglycerol by MprF and D-alanylation of lipoteichoic acids by DltABCD [65]. For *L. petauri* B1726, we identified homologous genes for GraRS (*lgb_00457* and *lgb_00458*), MprF (*lgb_01471*) and DltABCD (*lgb_01206-lgb_01209*). Hence, these factors may help to reduce adsorption of the cationic bacteriocin garvicin Q. Based on our results, we propose that the antimicrobial action of garvicin Q involves three steps: (I) adsorption of the hydrophobic/positively charged peptide to the negatively charged bacterial cell envelope, (II) interaction of garvicin Q with IIC and IID subunits of group I PTS^Man^ membrane components, and (III) structural changes resulting in formation of a pore in the transmembrane region of the IIC subunits leading to the inability to maintain membrane potential.

Finally, heterologous expression of the predicted *garQICD* operon in *C.* *glutamicum* resulted in production of an antimicrobial compound with identical features as garvicin Q produced by *L. petauri* B1726. These results strongly suggest that the genes *garQICD* are responsible for production of garvicin Q and indicates that recombinant production of garvicin Q is feasible. Also, our data does not provide information in the requirements of individual genes of the operon for garvicin Q production. This could be achieved by generating and analysing knockout mutants in the native host or by expression of different combinations of the *gar* genes in a heterologous host. Recently, similar experiments were performed for pediocin PA-1, another class II bacteriocin with a similar operon structure. The native pediocin PA-1 operon comprises the genes *pedABCD* with *pedA* encoding for the bacteriocin, *pedB* for an immunity protein and *pedCD* for two proteins involved in transport and processing. In *C. glutamicum* as heterologous host, *pedACD* are required for production of pediocin PA-1 but *pedB* is dispensable, possibly because *C. glutamicum* does not have a PTS^Man^ and is thus resistant [63].

A significant loss in garvicin Q activity was observed after 24 h of cultivation of *C. glutamicum* CR099/pPBEx2-*garQICD*^*Cgl*^. Cultivation of the recombinant strain in CGXII without urea markedly improved the production of the peptide and this is associated with acidification of the SN. Garvicin Q activity in SNs of the natural producer *L. petauri* B1726 is highest in late exponential to stationary phase when pH is highly acidic (pH 4–5; Fig. 4). Similar observation were made for other bacteriocins [78]. It is thus possible that acidic pH favourably impacts on its activity, stability, and/or adsorption to the producing cells.

Regarding efficiency of garvicin Q production using natural and recombinant hosts, it is difficult to compare the levels of activity and thus of the product garvicin Q obtained with *L. petauri* (natural producer) and *C. glutamicum* (heterologous host). There are differences in media (and potential interference with media components) as well as the sensor strains and the assays used for activity measurements. For heterologous production, an antibiotic (kanamycin) is required to maintain the plasmid. Thus, unlike for the natural producer strain, the sensor used to quantify activity has to carry a corresponding resistance to avoid measuring activity of the antibiotic instead of the bacteriocin. When simply comparing the dilutions required to detect garvicin Q activity it can be estimated that garvicin Q levels are at least fourfold higher in SNs of the natural producer *L. petauri* B1726 compared to the recombinant *C. glutamicum* producer despite lower final optical densities. This leaves ample room for improvement of recombinant garvicin Q production. Adsorption assays revealed that > 90% of the activity of purified garvicin Q was lost upon incubation with *C. glutamicum* despite the lack of a specific receptor, i.e. a PTS^Man^. This indicates that actual levels of garvicin Q are much higher, but the bulk of the peptide is not released into the SN and removed with the bacterial cells before activity assays. Further studies to understand (and prevent) the adsorption to bacterial cells may help to improve recombinant production of garvicin Q and other bacteriocins with *C. glutamicum* and provide an alternative to production using pathogenic, natural producers *L. garvieae* and *L. petauri*.

## Conclusion

We could demonstrate that *L. petauri* B1726 produces garvicin Q and optimized cultivation conditions for production of this bacteriocin. The genes responsible for biosynthesis of garvicin Q were identified in silico and their functionality was proven by heterologous expression in *C. glutamicum*. The presented data confirm the hypothesis that garvicin Q utilizes a PTS^Man^ as a receptor and provide evidence that it kills target bacteria by disruption of membrane integrity potentially by locking the PTS^Man^ into a conformation that leads to formation of a constitutively open pore. Moreover, we show that garvicin Q adsorbs to different types of bacteria independent of the presence of a group I PTS^Man^. Finally, we explore first avenues for the recombinant biotechnological production of garvicin Q.

## Supplementary Information

**Additional file 1.** Data file with supplementary Figures and Tables S1-14.

**Additional file 2.** Complete data set of QTOF LC-MS analysis of garvicin Q purified from supernatants the natural producer *L. petauri* B1726.

**Additional file 3.** Complete data set of QTOF LC-MS analysis of garvicin Q purified from supernatants the recombinant producer C. glutamicum CR099/pPBEx2-*garQICD*^Cgl^.

## Data Availability

All data generated or analysed during this study are included in this published article. Genome sequence of L. petauri B1726 is publically available on GenBank (Accession Number: CP094882.1). Raw sequencing data is available on the NCBI Sequencing Read Archive with accession numbers SRR18554891 (ONT data) and SRR18554892 (Illumina data).
