# Supplementary material for: Garvicin Q: characterization of biosynthesis and mode of action
Source: Microb Cell Fact. 2022 Nov 11;21:236. doi: 10.1186/s12934-022-01952-9 (PMC9652874; doi:10.1186/s12934-022-01952-9)
Supplement: Supplementary file 1 — Additional file 1. Data file with supplementary Figures and Tables S1-14. [file 12934_2022_1952_MOESM1_ESM.pdf]

# Supplementary data: Garvicin Q – characterisation of biosynthesis and mode of action

**Table S1: Primer and codon optimized gene sequences used in present study.**

| Primer | Sequence (5´->3´) | Purpose |
|---|---|---|
| **garQgarI-fwd-GA** | ATTACGCCAAGCTTGCATGCCTGCACTGCAGGAAGTCCAGGAG | Gibson assembly pPBEx2-*garQICD*$^{Cgl}$ |
| **garQgarI-rev-GA** | GGACTTCGGTACCGTCGACTTAGTCGAGATACG | |
| **garC-fwd-GA** | CGACTAAGTCGACGGTACCGAAGTCCAGGAG | |
| **garC-rev-GA** | GGACTTCGAGCTCGGTACCTTACTGGTTGTAGATC | |
| **garD-fwd-GA** | CCAGTAAGGTACCGAGCTCGAAGTCCAGGAG | |
| **garD-rev-GA** | CGGCCAGTGTATTCGAGCTCGGTACGAGCTCTTAATTCAGAGAATCTTG | |
| **garQI-seq** | CGGTTCTGGCAAATATTCTG | Sequencing of pPBEx2-*garQICD*$^{Cgl}$ |
| **garC-seq1** | GACGGATAGATGCCAGTG | |
| **garC-seq2** | GGTACTACAATTGAGGGTACTTCC | |
| **garC-seq3** | CAGGCGAATTCCATTCTGAATTCC | |
| **garC-seq4** | ACTTCTGTCTCCAGCCAAGATCC | |
| **garD-seq1** | CAGCTCAGTATCCTCCAACAATC | |
| **garD-seq2** | CGTCAGGATGGCCTTCTGC | |
| **Gene** | **Sequence (5´->3´)** | **Size [bp]** |
| ***garQ*$^{Cgl}$** | ATGGAGAACAACAACTACACCGTGTTGTCCGATGAAGAGCTTCAGAAGATCGATG<br>GAGGCGAATACCACCTGATGAATGGTGCCAATGGCTATCTCACTCGTGTCAACGG<br>CAAGTACGTCTATCGCGTTACCAAAGACCCAGTTTCCGCGGTATTCGGCGTGATT<br>AGCAACGGTTGGGGATCTGCTGGTGCAGGGTTTGGCCCTCAACATTAA | 213 |
| ***garI*$^{Cgl}$** | ATGTTTACCTCCAAGAAGATCAAACGCGATGAACAGGTGTTCCTGAACCTTCTGTA<br>CGACTTCGTGCTTTCTGAGAACATCACTGATCGTGAACGGAAAATCGGCTTGTTG<br>GCCAAGAAGGACGTAGAGAACGGCAAATACCTCCTTGCAGTCCTGAACAAGGTTA<br>GCTCATCCATGCAAAAGGAAGCGATGAAGAATGGTCTGTCCATTGATGCATCCAG<br>CTTCTACAAGAAACTGGGTCCAATCATCACCTCCATTGCTCCTATTGGGCTGAATC<br>GCGGATCTATGCTCGTCAACAACTCGTATCTCGACTAA | 315 |
| ***garC*$^{Cgl}$** | ATGCGGTTCAAGAAGAAGTACTATACCTCCCAAGTGGATGAGCGCGACTGTGGTT<br>GCGCAGCACTGTCCATGGTGCTCAAAACGTATGGAACCGAACAGTCACTGGCAT<br>CTATCCGTCTGTTGGCTGGTACTACAATTGAGGGTACTTCCGCTCTGGGAATCAA<br>GAAGGCTGCTGAACACCTGAAGTTCTCCGTGCAAGCCTTTCGCGCAGACCCAAC<br>ACTCTTCGATACCAAGAAGTTGCCTTACCCGTTCATCGTCCATGTCGTCAAAGAGA<br>AGAAATACCCTCACTACTACGTTGTCAAGTCGTCGAACAAGAAACACATTATCATC<br>GCAGATCCAGACCCTACCGTCAAGATCCGTAAAATGACCTGGGACCAGTTTTACT<br>CCGAGTGGACAGGCGTAACCTTGTTCATCGCACCACAGCCATCCTACAAACCCGT<br>GAAGGAGAAGTCCGAATCTTTGTTCTCGTTCATCCCAATCCTTGCTAAGCAGAAG<br>CTGCTGGTGTTCCAGATCATTATTGCGTCCCTTCTGGTTACACTGATTAACATCAT<br>TGGCAGCTACTACCTCCAAAACATCATCGACGAGTATGTCCCCAATGCCCTGATG<br>AAAACTCTGAGCATCGTGTCGCTCGGTCTGATGGCTACGTACTTGATTCAGCAGT<br>TCCTCAACTTTGCGCAGACCTTTCTCCTGACTATCCTGGGGCAACGTTTGGCTAT<br>CGACGTTATCCTTTCATACATCCGGCACATCTTTCAGCTCCCGATGTCCTTCTTTT<br>CGACCCGACGTACGGGTGAAATCACTAGCCGCTTTTCAGATGCGAACTCCATCAT<br>CGATGCGTTGGCTAGCGCTATTCTGTCCCTCTTTCTCGACGTAACCATCGTTATTC<br>TGACTGGCTTGGTTCTGGGTTTGCAGAACATGTCTCTCTTCTTCCTGGTGCTTTTC<br>AGCATTCCGCTCTATGTCCTTATCATCTTTATCTTCATTCCGTTGTTCGAGAAGCA<br>GAACAACGAAGTCATGCAGGCGAATTCCATTCTGAATTCCTCAATCATCGAGGAC<br>ATCAACGGTATCGAAACCATCAAATCCCTGGGCTCTGAAGAGATTCGCTATCAGA<br>AAATTGATCGCGAATTCGCCGATTACATGAAGAAATCGTTCACGCGCCAGAAGTC<br>GGAGGCATTCCAGACTGCCCTGAAGTCCGGTCTGCAACTGGTTCTGAACGTGTT<br>GATTCTTTGGTACGGTGCCACTCTGGTGATGTCCCAGAAAATCACCCTCGGACAA<br>CTTATCACCTTCAATGCTCTTCTGTCCTACTTCATGACCCCACTTTCTAACATCATC<br>AATCTCCAGACCAAGCTCCAATCTGCCAAGGTCGCGAACCATCGGCTGAATGAAG | 2148 |

| | | |
|---|---|---|
| | TTTACCTGGTGGATAGCGAGTTTGACACGAACACCGATGAGGTTATCCTTTCCAA<br>CTTTGAGATGCAGCTGATCGGCATTAGCTATCACTACGGCTTTGGGCGTGACATT<br>CTTTCTAACATGTCCCTTAACATCAAAGAAAACGAAAAGCTTACCATTGTCGGAAT<br>GTCTGGCTCAGGCAAATCCACCCTCGTTAAGCTTCTCGTAAACTTCTTCGAACCC<br>ACCCAAGGAACCATTATCCTGGGCGGGATTGACATCAAACAAATGGATAAACACC<br>AGCTGCGTTCTATCGTGTCATATTTGCCTCAACAGCCTTACATCTTCACCGGCACC<br>GTCTTGGAGAACCTTATTCTGGGCGCGTCTGAAGATCTCTCTCAGGAAGAGATTT<br>TCCAGGCAGTAGAGGCAGCAGAAATCCGATCCGATATCGAACAGCTCCAACTGG<br>GCTATCAGACGGAGCTGTCATCCGATGCCACAACTCTCTCGGGTGGACAGAAGC<br>AACGCATTGCGCTCGCTCGCGCACTTCTGTCTCCAGCCAAGATCCTGATCTTCGA<br>TGAGGCTACCAGCAATCTGGACGTTATTACCGAGAAGAAGATTTTGAAGAATCTCT<br>TCAAACTGGACAAGACCATCATCTTCATCGCCCATCGCTTGAGCATTGCAGAAAA<br>GTCCGACCGCATCATTGTGATCGATCAGGGGAAAATCATGGAAGAAGGCTCCCA<br>CTTGGAACTGCTTTCCAAGAATGGCTTCTACGCCCAGATCTACAACCAGTAA | |
| ***garD$^{Cgl}$*** | GTGTTTGATAAGCGACTCTTGGAGTCGTCTGAGCTGTATGAAAAGCGCTACAAGA<br>ATTTCTCCGTCCTCCTCATCTTTCCAGTCGCACTGTTGTTTATCGGTCTGTTCGTG<br>TTCAGCTTCTTCGCTAAGAAGGAGCTGATCGTGACGAACATTGCCTCCATTGCTC<br>CGAAGAAGATCATCAGCAACATTCAATCCACCTCCAATACGCCCATTATCGAAAAC<br>CACCTGGTTGAGGGGAAATCAGTGCAGTCTAATTCACTGCTCATCAAGTACAACA<br>ACGACTCTGACTCTACTCAGATTACCACCCTGATGGATCAAAAGCAGGAATTCCT<br>CAACAAGAAGGAGCAACTTCAGTTGCTTCTGAATTCGCTGCACTCTGATACCAAC<br>CAGTTCCCAAACACCGATTCCTATGGATACGAAAAGACGTTCGAAACCTACAAGG<br>CACAAGCCGAATCCCTTCGCGAATCGATCCAGAAATCGAACCAAGTGGTTGACGA<br>TCAGAACAACTCCATTGAGAACCAGAAGTCTGCAATCACCACGCAGATTTCCAAC<br>GTAAACACCCAAATCAATGCTTACATCGACATCCAGAATGCAGCTAGCACCAACA<br>GCTCAGTATCCTCCAACAATCCGTACATCGCTCAGTATAACTCGTACTTGGAACAG<br>CGCAAGGTTCTCGAAGAATCCATGAAGAATCAGGAAAAGACTGAAGCCTCCTCAA<br>AGGCCGACATTACCCAGCAGAAAGAGGCCTTGAAAGCGCAATTCATCTCCAACAT<br>CTCCTCCAACATTGACAACCTTAAGAATCAGATCCAGACACTGGAGGTCCAGAAA<br>TCCTCCCTGAGCAGCTCCAACTCTTACGATCAATCTCAGAAAAGCCAACTCCTCG<br>CGCTTAAAACTCAGGCGCTCACTGCTGCGAACAAAGAACTGTCCGACATCAATAG<br>CACACTTACCGAGATTGGTGGCAAAATCTCTCTCCAGAAACAGGCCAACCAATAC<br>AACGCAATCTTTGCTGAGAAAGAGGGTATCTTGCATGTTCTGCCTAACGTGCTGG<br>AGAACAAAACCTTCCAGGTAGGTACTCCACTGGCACAGATCTATCCTGCTCTGAA<br>GGCGAAGTCACAGGTCTATCTGACTTCATACATTCCCTCCACCCAGATTTCAGGC<br>ATTAAGCTTGGACAGCGTGCACGCTTCACGGTGCAGCAAAACTTGCCTAAGCTTG<br>AGATCCTGACTGGAACCATCAAGCAGATCGATTCCGCACCAACAGCCCTTAAGGA<br>GGGTAATTCGTACAAAGTGTCGGCACTGGTTACCTTGAACGAACGTGATCTCTCC<br>TACATCCGGTATGGCTTGGAAGGCAAGTTTGTCGTCGTTACCGGCCAAAAGACCT<br>ACTTCAACTACTACCTGGACAAAATCAAGGGCCAAGATTCTCTGAATTAA | 1428 |

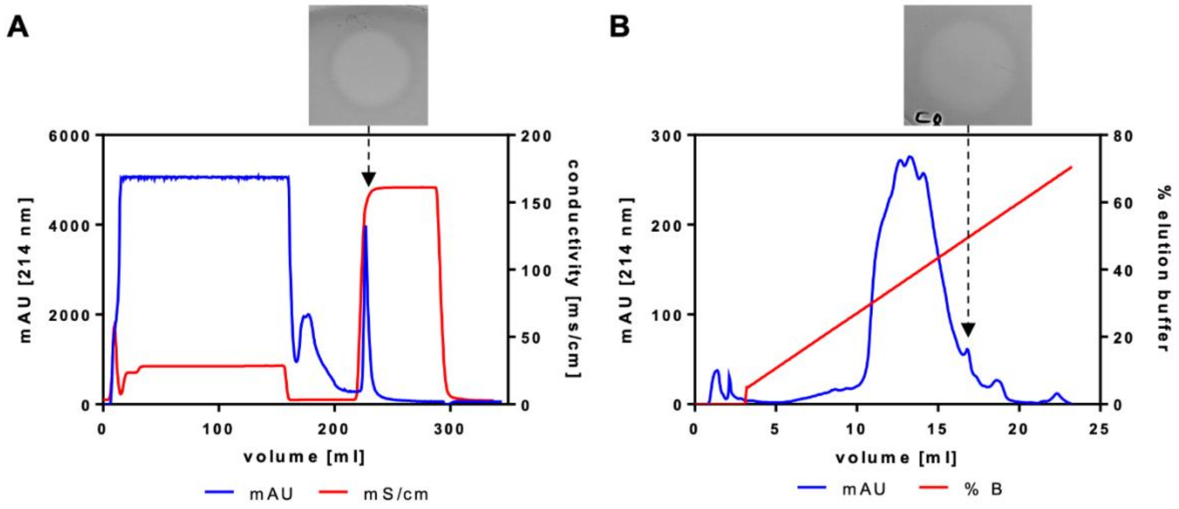


**Figure S2: Concentration of *Lactococcus* sp. B1726 peptide by cation exchange (CIEX) and reverse-phase chromatography (RPC).** CIEX of ammonium sulphate precipitation of cell-free supernatants of *Lactococcus* sp. B1726 (A) and RPC of the active CIEX elution fraction (B). The black arrow indicates the active fraction. Antimicrobial activity was tested by spot on lawn assay with *L. innocua* LMG2785 as sensor strain.

**Table S3: Whole genome sequence alignment of *Lactococcus* sp. B1726 to other *Lactococcus* species.** JSpeciesWS was used for performing the alignment. A tetra correlation search for Isolate B1726 was done to find similar genomes. Identified genomes were compared by ANIm (average nucleotide index based on MUMer). The percentage of aligned nucleotides is also indicated as well as the source, safety level and potential bacteriocins.

| Genome | ANIm [%] | Aligned [%] | source | safety level | putative bacteriocin (BAGEL4) |
|---|---|---|---|---|---|
| *Lactococcus garvieae* PAQ102015-99 | 99.94 | 97.39 | rainbow trout | 2 | no |
| *Lactococcus* sp. DD01 | 98.57 | 88.44 | nasopharynx/oral cavity of primates | no data | yes (lactococcin/enterocin like) |
| *Lactococcus petauri* 159469 [T] | 98.55 | 90.99 | abscess on a sugar glider | no data | no |
| *Lactococcus garvieae* M14 | 98.24 | 85.96 | no data | 2 | yes (garvicin Q gene) |
| *Lactococcus garvieae* Lg-ilsanpaik-gs201105 | 98.17 | 86.17 | human gall bladder | 2 | no |
| *Lactococcus garvieae* IPLA 31405 | 93.73 | 84.53 | spanish traditional cheese Casin | 2 | yes (garvicin Q) |
| *Lactococcus garvieae* Lg2 | 93.33 | 80.73 | no data | 2 | no |
| *Lactococcus garvieae* ATCC 49156 [T] | 93.30 | 80.95 | no data | 2 | no |
| *Lactococcus garvieae* TRF1 | 93.23 | 83.73 | Fecal material of timber rattlesnake | 2 | no |
| *Lactococcus formosensis* NBRC 109475 [T] | 92.95 | 82.92 | No data | no data | no |
| *Lactococcus lactis* subsp. *lactis* Il1403 IL1403 | 86.19 | 5.92 | Derivative of IL594 (cheese starter culture | 1 | no |
| *Lactococcus cremoris* P7266 | 85.14 | 5.38 | Litter on pastures | 1 | no |
| *Lactococcus taiwanensis* NBRC 109049 [T] | 85.09 | 5.35 | no data | no data | no |
| *Streptococcus agalactiae* BV3L5 | 85.04 | 1.76 | no data | 2 | yes (zoocin A and nisin U) |
| *Lactococcus garvieae* DCC43 | 84.86 | 45.44 | Mallard duck intestines | 2 | yes (garvicin ML) |
| *Streptococcus* sp. oral taxon 056 str. F0418 | 84.75 | 2.01 | no data | no data | yes (putative bacteriocin) |
| *Lactococcus fujiensis* JCM 16395 [T] | 84.58 | 3.74 | no data | no data | no |

[T] – Type strain

**Table S4: Predicted virulence factors and proteins important for survival in fish encoded on the genome of *L. petauri* B1726.** The annotated genome of *L. petauri* B1726 was searched for virulence factors or proteins importat for survival in the host described for pathogenic strains of *L. garvieae*.

| | Putative virulence factors | Function/explanation | Reference | Locus tag | COG number / EC number |
|---|---|---|---|---|---|
| **Adhesion** | Sortase A (*strA*) | transpeptidase | [1] | lgb_00676 | COG3764 / 3.4.22.- |
| | Internalin J (*inlJ*) | cell adhesion | [1] | lgb_01130 | - |
| | Enolase (*eno*) | surface adhesin | [1] | lgb_01499 | COG0148 / 4.2.1.11 |
| | PavA-like protein (*rqcH*) | surface adhesion, fibronectin-binding domain | [2] | lgb_00660 | COG1293 |
| | PsaA-like protein (*mntA*) | surface adhesion, Manganese-binding lipoprotein MntA | [2] | lgb_01517 | COG0803 |
| **Cytotoxicity** | Hemolysin A (*tlyA*) | α-haemolysis | [1] | lgb_00580 | COG1189 |
| **Survival/stress response** | Superoxide dismutase (*sodA*) | tolerance to aerobic environments | [1] | lgb_00290 | COG0605 / 1.15.1.1 |
| | NADH oxidase (*noxE*) | tolerance to aerobic environments | [1] | lgb_01366 | COG0446 / 1.6.99.3 |
| | N-acetyltransferase Eis | May enhance intracellular survival | [3] | lgb_00930 | 2.3.1.- |
| | Delta subunit of RNA polymerase (*rpoE*) | Global modulator of environmental adaptation | [4] | lgb_00356 | - |
| **Metabolism** | Asparagine synthetase (*asnA*) | Asparagine synthesis | [3] | lgb_01041 | COG2502 / 6.3.1.1 |
| | α-acetolactate synthase | involved in the biosynthesis of acetoin and 2,3-butanediol | [3] | lgb_01091 | COG0028 / 2.2.1.6 |
| | Polyamine ABC-transporter (*potABCD*) | Polyamine uptake | [4] | lgb_00916-lgb_00919 | 7.6.2.11 (PotA), COG1176 (PotB), COG0687 (PotD) |
| **Cell envelope modification** | D-alanine-D-alanyl carrier protein ligase (*dltA*) | D-alanylation of teichoic acids | [3] | lgb_01026 | COG1020 / 6.2.1.54 |
| | Phosphoglucomutase (*pgcA*) | resistance to peptide antimicrobials | [1] | lgb_01633 | 5.4.2.2 |
| **Unclear** | DNA processing protein DprA | unknown | [3] | lgb_01056 | COG0758 |
| | *xerC* | Tyrosine recombinase | [3] | lgb_00045, lgb_00697, lgb_01540, lgb_02004 | - |

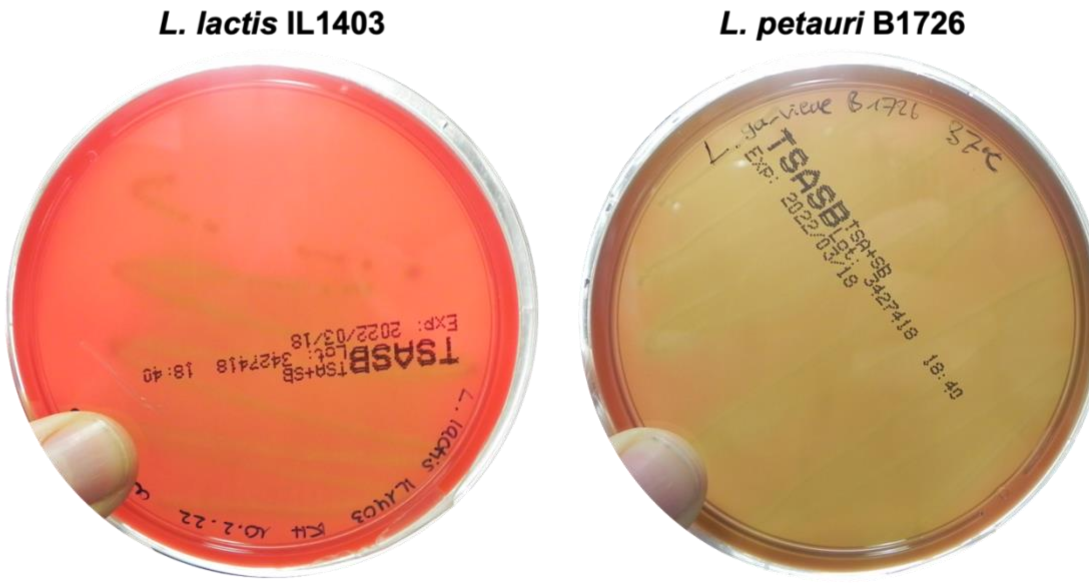


**Figure S5: α-hemolysis by *L. petauri* B1726.** *L. petauri* B1726 and *L. lactis* IL1403 were streaked on tryptone soya agar supplemented with 5 % sheep blood (Thermo Scientific™). The agar plates were incubated at 37 °C. Incubation of *L. petauri* B1726 for ~4 days led to a clear discoloration of the blood agar indicating α-hemolysis.

ACTTAACCTTTTCTTAA***TTGTTA***TCAAAAACACAAAG***TAGTATAAT***ATATTTGTTTAAGTAAAAGTGTTTGAATATTTTAGAATAAGGAGAAAAAAATA
TGGAAAACAACAATTACACAGTCCTTTCAGATGAAGAACTACAAAAAATTGATGGTGGAGAATACCATCTAATGAACGGGGCTAATGGTTATTTAACG
AGAGTAAATGGGAAATATGTATATCGAGTTACAAAGGATCCCGTTAGTGCAGTCTTTGGAGTTATCTCAAACGGCTGGGGAAGCGCTGGGGCTGGTTT
TGGTCCCCAACATTAATCTTAATAGAAACGAGATATTTCATGTTTACTTCTAAAAAAATTAAACGAGATGAGCAAGTTTTTTAAACTTATTATATGA
TTTTGTTTTAAGCGAAAATATCACAGATAGAGAGAGAAAAATTGGATTACTAGCCAAAAAAGATGTGGAAAATGGAAAGTATTTACTGGCAGTGTTAA
ATAAGGTATCTTCAAGCATGCAAAAAGAAGCAATGAAAAATGGATTATCTATTGATGCTTCTTCTTTTTATAAAAAACTTGGCCCTATTATTACAAGT
ATTGCCCCAATTGGTTTAAATAGAGGTTCTATGTTGGTTAACAATAGTTACTTAGACTGATTATATTACAAATTATCTTCATCATTTCACT***TTTTAAA***
***TT***ATAAAAGATGAGAAATATATTCTCATCTTTTATATTGTCAACGAT***TTATCT***TAAAAGTTAGTGAATCAAAAAAACAGGAAGAAATTATGCGTTTTA
AAAAGAAATATTATACGTCACAAGTGGACGAAAGAGATTGTGGGTGTGCTGCTCTTTCCATGGTTTTAAAAACTTATGGTACAGAACAATCCTTAGCT
TCAATACGTTTACTTGCTGGTACAACGATAGAAGGCACTTCTGCTTTAGGTATAAAAAAAGCCGCGGAACATTTAAAGTTCAGCGTTCAAGCCTTTCG
TGCAGATCCAACTCTATTTGATACAAAGAAACTTCCTTATCCATTTATTGTTCATGTGGTTAAAGAAAAGAAATACCCTCACTATTATGTGGTTAAAT
CCTCCAATAAAAAACATATCATTATTGCAGATCCAGATCCCACGGTTAAAATTCGGAAAATGACTTGGGACCAGTTTTATTCTGAATGGACAGGTGTA
ACTTTATTTATTGCTCCTCAACCTTCTTATAAACCAGTGAAAGAAAAATCCGAGTCTTTGTTTTCTTTTATACCCATACTCGCAAAGCAAAAGTTGCT
TGTTTTTCAGATTATTATTGCTTCTCTTTTGGTCACTTTAATTAATATTATCGGCTCTTATTATCTTCAGAATATTATTGACGAATATGTGCCTAATG
CTTTAATGAAGACTTTAAGCATTGTTTCTCTCGGATTAATGGCAACCTATCTCATCCAGCAATTCCTTAATTTTGCTCAAACTTTCTTACTTACAATA
CTTGGACAACGTTTAGCCATTGATGTTATCCTTTCCTATATCCGTCATATTTTTCAACTTCCTATGTCCTTTTTTTCTACACGAAGAACGGGAGAAAT
TACCAGTCGTTTCTCTGACGCTAATTCAATTATTGATGCCCTTGCTTCTGCCATCTTATCTCTTTTCTTAGATGTCACGATTGTCATTCTGACAGGGC
TTGTTTTAGGACTTCAAAACATGAGCTTATTCTTCCTTGTCTTGTTTTCCATTCCACTTTATGTTCTTATCATTTTTATTTTTATTCCTTTATTTGAA
AAACAAAATAATGAAGTCATGCAAGCAAATTCTATTTTAAATTCATCAATTATTGAAGATATCAATGGAATTGAAACCATAAAATCTCTTGGGAGTGA
AGAGATTCGCTATCAAAAAATAGACCGTGAATTTGCGGACTACATGAAAAAATCATTCACGCGTCAAAAATCCGAAGCTTTTCAAACTGCCTTGAAAT
CAGGATTACAACTTGTTTTAAATGTTCTTATCTTGTGGTATGGAGCCACCCTCGTGATGAGCCAAAAAATCACCTTAGGTCAATTGATTACTTTCAAT
GCCTTACTTTCATATTTTATGACTCCTCTCTCTAATATCATCAACTTACAAACTAAACTTCAATCCGCTAAAGTAGCTAACCATCGTTTAAATGAAGT
TTACTTGGTAGACAGTGAATTTGATACAAATACAGATGAAGTTATTCTTTCAAATTTTGAGATGCAGTTGATAGGTATATCTTACCACTATGGTTTTG
GTAGAGACATTCTCTCTAATATGTCACTTAATATAAAAGAAAATGAAAAATTAACAATTGTCGGAATGAGTGGCTCAGGTAAAAGTACCCTAGTCAAA
TTGTTGGTTAATTTTTTCGAACCTACACAAGGGACTATCATTCTTGGGGGGATAGATATAAAACAAATGGATAAGCACCAATTGAGAAGTATTGTCAG
CTATCTTCCCCAACAACCGTATATCTTTACAGGAACAGTACTAGAGAACTTAATCTTAGGAGCAAGTGAGGATCTTTCTCAGGAAGAAATTTTCCAAG
CTGTTGAAGCTGCAGAAATTCGTTCCGACATTGAACAGCTTCAATTGGGCTATCAAACAGAACTATCTAGTGATGCTACAACTCTCTCTGGAGGGCAA
AAACAAAGAATCGCATTGGCTCGTGCCTTACTTTCTCCCGCGAAGATTCTTATTTTTGATGAAGCAACAAGTAATCTGGATGTAATTACCGAGAAAAA
AATTTTGAAAAATCTATTTAAGTTAGATAAAACCATTATTTTTATTGCCCATCGTCTGTCTATCGCGGAAAAGAGCGACAGGATTATTGTTATTGATC
AGGGAAAAATTATGGAAGAAGGGAGTCATTTAGAACTGCTTTCTAAAAATGGTTTTTATGCACAAATTTATAATCAATAGGAGGTTCACGTGTTTGAT
AAACGTTTATTAGAGAGTTCAGAACTCTATGAAAAGAGATATAAAAATTTCTCGGTACTTCTTATATTTCCAGTAGCTCTTCTTTTTATTGGATTGTT
TGTTTTTTCTTTCTTTGCAAAA**AAAGAATTG**ATTGTCACTAATATTGCAAGTATTGCCCCCAAAAAAATTATTTCTAATATCCAGTCGACAAGTAATA
CACCTATTATTGAGAATCATCTCGTAGAAGGCAAATCAGTTCAATCTAATAGCTTACTTATAAAATATAATAATGACTCGGACAGCACGCAAATCACA
ACCTTAATGGATCAGAAGCAAGAGTTTTTAAATAAAAAAGAACAACTTCAACTTCTTTTGAATAGTCTTCACTCAGATACCAATCAATTCCCTAATAC
AGATTCTTACGGCTATGAAAAAACATTCGAAACATATAAAGCTCAAGCAGAGAGCTTAAGAGAAAGCATACAAAAGTCTAACCAAGTTGTAGACGATC
AGAACAACAGCATTGAGAATCAGAAGTCGGCTATTACTACACAAATTTCTAATGTTAACACTCAAATTAATGCTTATATTGATATCCAAAATGCAGCA
TCTACAAATAGCTCTGTATCCTCAAATAACCCTTATATAGCCCAGTATAATAGCTACCTTGAACAACGAAAAGTTCTTGAAGAGAGCATGAAAAATCA
AGAAAAAACAGAAGCTTCCTCTAAAGCAGATATCACTCAACAAAAAGAAGCTTTAAAAGCTCAATTTATATCCAACATCTCTTCAAATATTGATAATT
TAAAAAATCAAATACAAACTTTAGAAGTACAAAAAAGTAGTTTAAGCTCAAGCAATAGCTATGATCAAAGTCAAAAGAGCCAACTCCTCGCTCTTAAA
ACACAAGCTCTAACTGCTGCAAATAAAGAACTATCTGATATAAATAGTACACTCACCGAAATAGGTGGTAAAATTAGCTTACAAAAACAAGCCAATCA
ATACAATGCAATTTTTGCGGAAAAGGAGGGCATTTTGCATGTTTTACCTAATGTTCTAGAAAATAAAACATTCCAAGTAGGCACTCCACTCGCTCAAA
TTTATCCCGCATTAAAAGCTAAATCTCAAGTTTATTTAACCTCTTATATTCCAAGTACTCAAATATCTGGTATCAAGTTAGGACAAAGAGCTCGCTTT
ACTGTTCAACAAAACCTTCCTAAGCTAGAAATATTAACGGGAACTATTAAACAAATTGATAGCGCGCCAACAGCTTTAAAGGAAGGCAATAGTTATAA
GGTTTCTGCACTCGTTACCTTGAATGAAAGAGACCTCTCTTATATTCGATATGGTTTAGAAGGTAAATTTGTAGTAGTTACTGGTCAAAAGACTTATT
TTAACTATTATTTAGATAAAATAAAAGGACAAGATTCGTTGAATTAAACTCGAAGACATAAAGTAAGAACAGCACTCTCTGTATTTTAGAGAGTGCTA
TTTTATAGTCATTGCAAAATAATT

**Figure S6: Garvicin Q operon of *L. petauri* B1726.** Nucleotide sequence of *garQ* (red), *garI* (blue), *garC* (orange) and *garD* (green) are given. Predicted promoter sequences (-10 and -35 boxes) are displayed in italic and bold letters. Rho-independent terminator sequences are underlined. A putative ribosome binding site and start codon

within *garD* is highlighted by capital and bold letters. FGENESB and BPROM were used for prediction of promoter and transcription units. ARNold was used for finding terminators.

**Table S7: Impact of different growth conditions on garvicin Q production by *L. petauri* B1726.** *L. petauri* B1726 was cultivated in 5 ml medium in glass tubes overnight. The antimicrobial activity was determined by growth inhibition assay with *L. innocua* LMG2785 as sensor strain.

| **Medium** | **$OD_{600}$** | **pH** | **BU/ml** |
|---|---|---|---|
| MRS, 25 °C, static | 2 | 4.8 | 320 |
| MRS, 30 °C, static | 2.4 | 4.8 | 640 |
| MRS, 37 °C, static | 2.3 | 4.6 | 160 |
| MRS, 30 °C, shaken | 1.0 | 5.5 | 160 |
| MRS, 30 °C, anaerobic | 2.1 | 4.8 | 640 |
| M17 [2 % glucose] 30 °C, static | 2.8 | 4.4 | 1280 |
| M17 [2 % lactose] 30 °C, static | 0.7 | 6.8 | 0 |
| BHI [2 % glucose] 30 °C, static | 2.4 | 4.2 | 160 |
| 2xTY [2 % glucose] 30 °C, static | 1.6 | 4.2 | 0 |

**Table S8: Ammonium sulphate (AS) precipitation of garvicin Q.** Cell-free culture supernatant of an overnight *L. petauri* B1726 culture was subjected to AS precipitation applying different AS concentrations. The precipitate was dissolved in demin $H_2O$ (dissolved precipitate). The dissolved precipitate was clarified by centrifugation to remove insoluble components and the clarification supernatant was used in a growth inhibition assay (clarification). The percentage of recovered activity after clarification compared to the dissolved precipitate was then calculated (recovered activity). The antimicrobial activity was determined by growth inhibition assay with *L. innocua* LMG2785 as sensor strain. Although the recovered activity was highest for 20-30 % of AS, the highest overall activity was determined in the dissolved precipitate using 70 % AS. These results indicate that >50 % of AS reduces solubility of garvicin Q.

| **AS concentration** | **20 %** | **30 %** | **40 %** | **50 %** | **60 %** | **70 %** |
|---|---|---|---|---|---|---|
| **dissolved precipitate [BU/ml]** | 160 | 1280 | 2560 | 2560 | 2560 | 5120 |
| **clarification [BU/ml]** | 160 | 1280 | 1280 | 320 | 320 | 320 |
| **recovered activity [%]** | 100 | 100 | 50 | 12.5 | 12.5 | 12.5 |

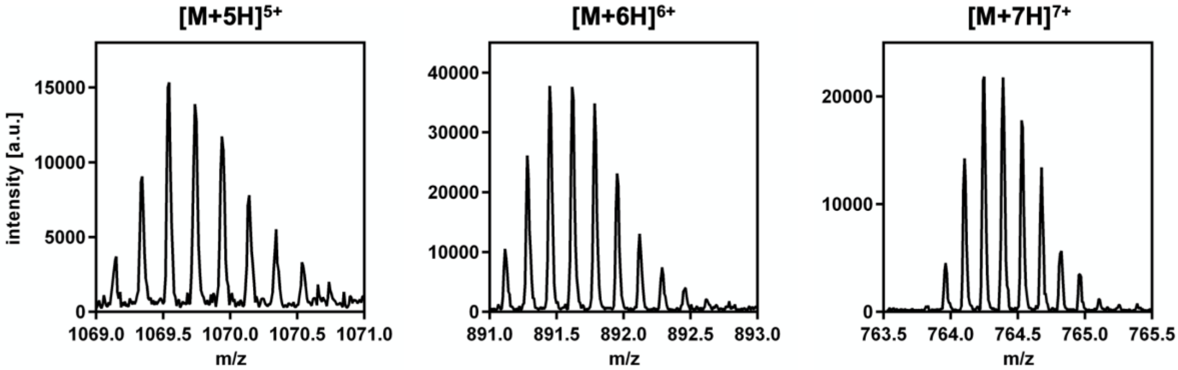


**Figure S9: QTOF LC-MS analysis of natural garvicin Q in pooled RPC elution fractions harbouring antimicrobial activity.** Graphs are derived from the spectrum shown in Figure 4D zoomed in on the peaks with a mass to charge ratio (m/z) of 1069.1500 (left), 891.1142 (middle) and 763.9628 (right), which correspond to the 5-fold ($[M+5H]^{5+}$; m/z spacing of 0.2), 6-fold ($[M+6H]^{6+}$; m/z spacing of 0.17), and 7-fold ($[M+7H]^{7+}$; m/z spacing of 0.14) positively charged ion of garvicin Q.

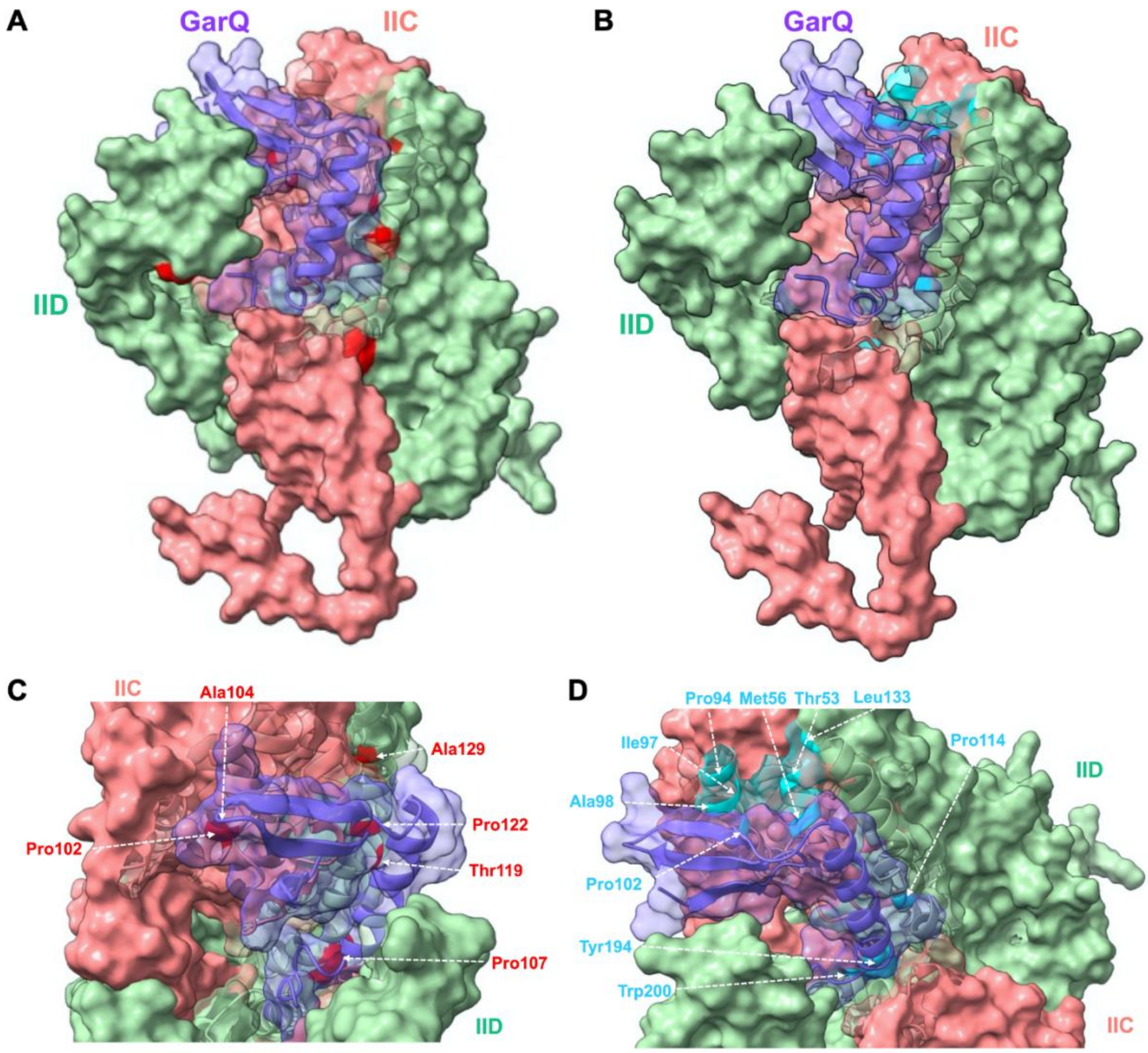


**Figure S10: Predicted interaction of garvicin Q with the IIC and IID subunit of the *L. monocytogenes* EGDe group I PTS$^{Man}$.** Garvicin Q (GarQ) is indicated in blue. The PTS$^{Man}$ subunits IIC and IID are indicated in pink and green. The surface of garvicin Q and PTS$^{Man}$ residues in close vicinity to garvicin Q (within 5 angstrom) is transparent. **(A)** and **(C)** Amino acid residues that were previously described to be involved in the interaction of garvicin Q and PTS$^{Man}$ by Tymoszewska et al., 2017 [5]are indicated in red. **(B)** and **(D)** Amino acid residues that were previously described to be involved in the interaction of pediocin and PTS$^{Man}$ by Zhu et al., 2022 [6] are indicated in cyan. AlphaFold was used for structure prediction and ChimeraX for visualization.

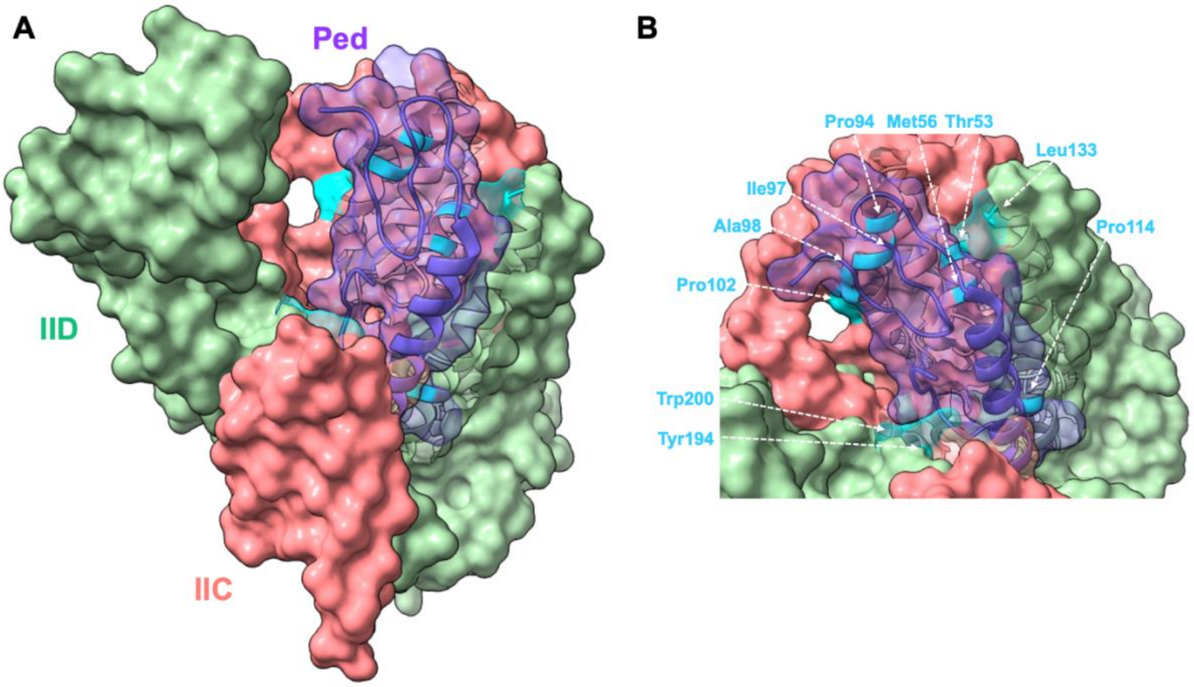


**Figure S11: interaction of pediocin PA-1 with the IIC and IID subunit of the *L. monocytogenes* EGDe group I $PTS^{Man}$ (PDB: 7VLY).** Pediocin PA-1 (Ped) is indicated in blue. The $PTS^{Man}$ subunits IIC and IID are indicated in pink and green. In **(B)**, amino acid residues that were previously described to be involved in the interaction of pediocin and $PTS^{Man}$ by Zhu et al., 2022 [6] are indicated in cyan. ChimeraX was used for visualization.

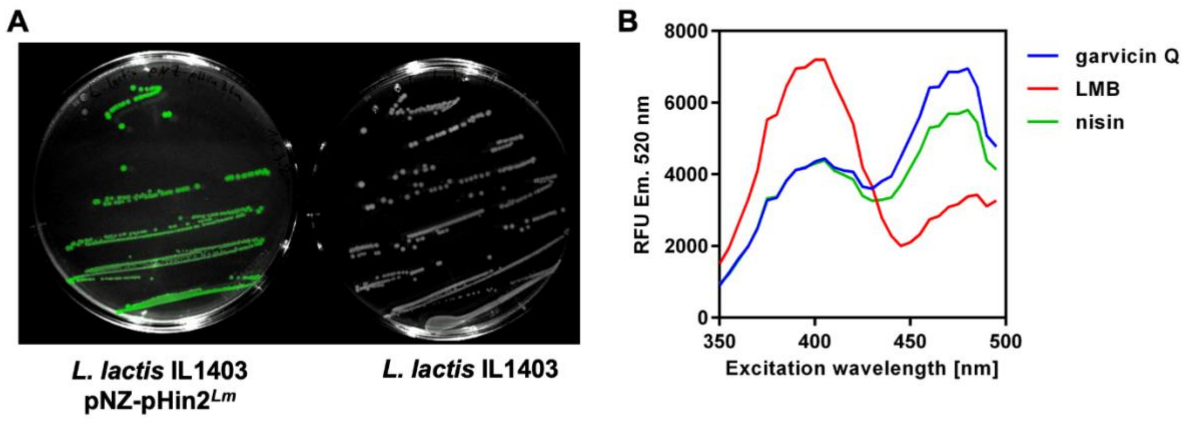


**Figure S12: Characterisation of *L. lactis* IL1403/pNZ-*pHin2*$^{Lm}$. (A)** Fluorescence imaging of the recombinant strain *L. lactis* IL1403 pNZ-pHin2$^{Lm}$ and the wild type strain. The recombinant strain showed fluorescence compared to the wild type strain. This indicates synthesis of pHluorin2 protein. Imaging was performed in an iBright$^{TM}$ FL 1000 imaging system with filter settings for green fluorescence. **(B)** Excitation spectrum of *L. lactis* IL1403 pNZ-pHin2$^{Lm}$*.* Addition of the pore-forming bacteriocin nisin (green) and garvicin Q (blue) is shifting the ratio of the excitation maxima in comparison to the LMB buffer negative control (red)

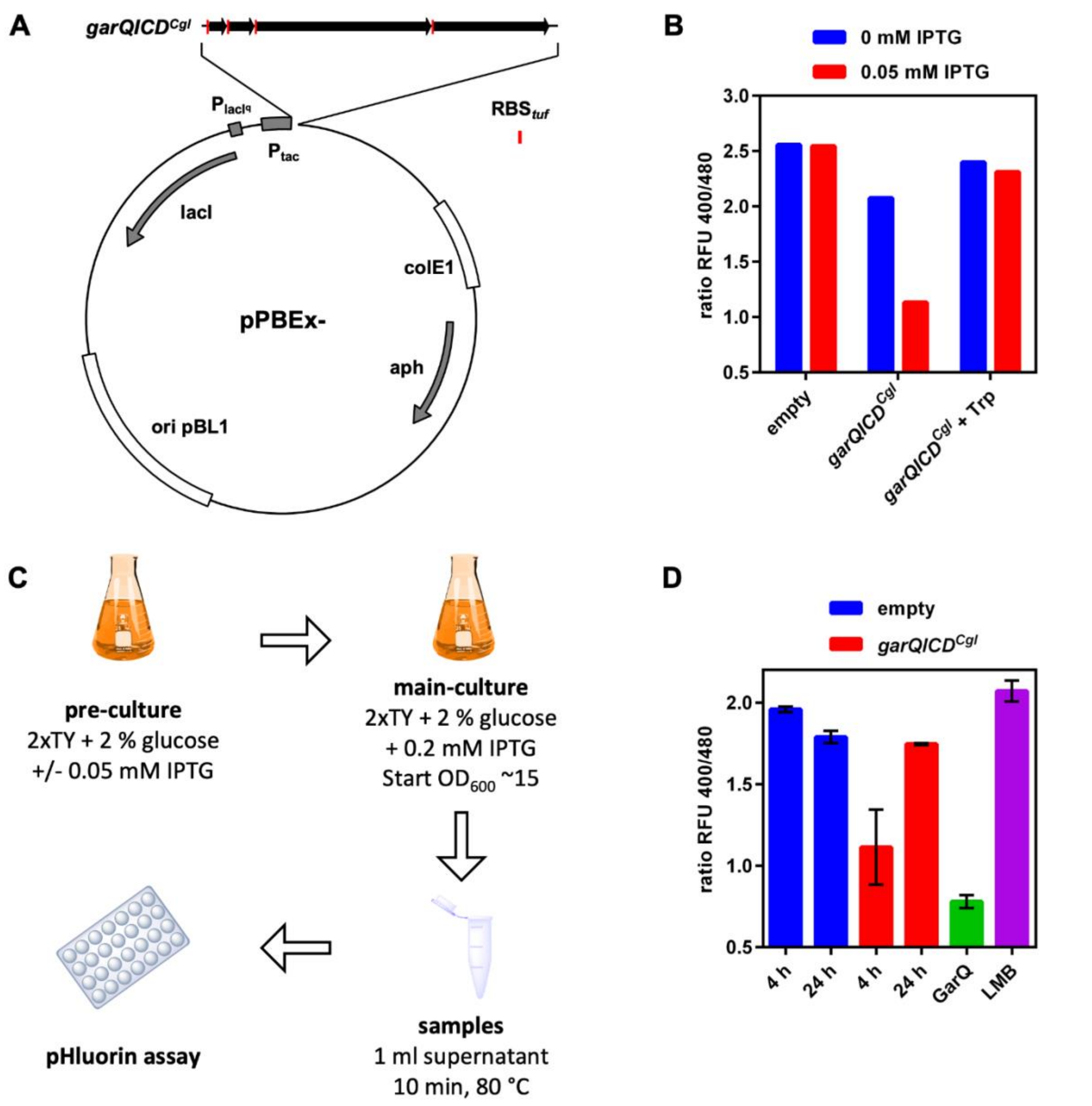


**Figure S13**: **Establishment of recombinant garvicin Q production by *C. glutamicum*. (A)** Schematic map of expression vector pPBEx2-*garQICD*$^{Cgl}$. Upstream of each gene the sequence of the ribosomal binding site (RBS) of the elongation factor TU was inserted**. (B)** Influence of induction of gene expression during pre-cultivation of *C. glutamicum* CR099/pPBEx2-*garQICD*$^{Cgl}$. Garvicin Q activity was determined by pHluorin2 assay using *L. lactis* IL1403/pNZ-pHin2$^{Lm}$ as a sensor. Samples were taken 4 h after inoculation of the main culture. Addition of 0.05 mM IPTG during pre-cultivation led to a more pronounced decrease in the fluorescence ratio indicating pore formation/garvicin Q activity. Trypsin treatment completely abolished antimicrobial activity indicated that the antimicrobial compound is a protein/peptide. **(C)** High-cell density cultivation of *C. glutamicum* CR099/pPBEx2-*garQICD*$^{Cgl}$ for garvicin Q production. **(D)** Activity in supernatants of *C. glutamicum* CR099/pPBEx2-*garQICD*$^{Cgl}$ or the control strain harbouring the empty plasmid pPBEx2 at 4 and 24 h of cultivation in a high-cell density approach. Assay were performed with *L. lactis* IL1403/pNZ-pHin2$^{Lm}$ as indicator. Assay buffer (LMB) served as negative

control and HIC purified garvicin Q (GarQ) was used as a positive control. Values in (B) and (D) are ratios of fluorescence intensity at 520 nm after excitation at 400 and 480 nm (ratio RFU 400/480) and are mean of n = 2-3 independent experiments (supernatants of independent cultivations).

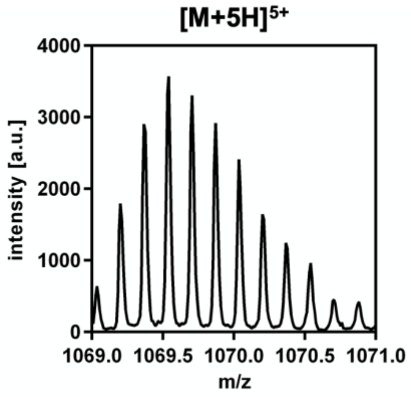


**Figure S14: QTOF LC-MS analysis of recombinant garvicin Q in pooled RPC elution fractions harbouring antimicrobial activity.** The graph is derived from the spectrum shown in Figure 7D zoomed in on the peak with a mass to charge ratio (m/z) of 1069.2010, which corresponds to the 5-fold positively charged ion of garvicin Q ($[M+5H]^{5+}$; m/z spacing of 0.2).

# Supplementary References

1. Gibello A, Galán-Sánchez F, Blanco MM, Rodríguez-Iglesias M, Domínguez L, Fernández-Garayzábal JF: **The zoonotic potential of Lactococcus garvieae: An overview on microbiology, epidemiology, virulence factors and relationship with its presence in foods.** *Res Vet Sci* 2016, **109:**59-70.
2. Morita H, Toh H, Oshima K, Yoshizaki M, Kawanishi M, Nakaya K, Suzuki T, Miyauchi E, Ishii Y, Tanabe S, et al: **Complete genome sequence and comparative analysis of the fish pathogen Lactococcus garvieae.** *PLoS One* 2011, **6:**e23184.
3. Menéndez A, Fernández L, Reimundo P, Guijarro JA: **Genes required for Lactococcus garvieae survival in a fish host.** *Microbiology (Reading)* 2007, **153:**3286-3294.
4. Aguado-Urda M, Gibello A, Blanco Mdel M, Fernández-Garayzábal JF, López-Alonso V, López-Campos GH: **Global transcriptome analysis of Lactococcus garvieae strains in response to temperature.** *PLoS One* 2013, **8:**e79692.
5. Tymoszewska A, Diep DB, Wirtek P, Aleksandrzak-Piekarczyk T: **The Non-Lantibiotic Bacteriocin Garvicin Q Targets Man-PTS in a Broad Spectrum of Sensitive Bacterial Genera.** *Scientific Reports* 2017, **7:**8359.
6. Zhu L, Zeng J, Wang C, Wang J: **Structural Basis of Pore Formation in the Mannose Phosphotransferase System by Pediocin PA-1.** *Appl Environ Microbiol* 2022, **88:**e0199221.
